# C‐X‐C motif chemokine receptor 4 aggravates renal fibrosis through activating JAK/STAT/GSK3β/β‐catenin pathway

**DOI:** 10.1111/jcmm.14973

**Published:** 2020-03-02

**Authors:** Yahong Liu, Qijian Feng, Jinhua Miao, Qinyu Wu, Shan Zhou, Weiwei Shen, Yanqiu Feng, Fan Fan Hou, Youhua Liu, Lili Zhou

**Affiliations:** ^1^ Division of Nephrology State Key Laboratory of Organ Failure Research National Clinical Research Center of Kidney Disease Nanfang Hospital Southern Medical University Guangzhou China; ^2^ Division of Nephrology The Second Affiliated Hospital of Xingtai Medical College Xingtai China; ^3^ School of Biomedical Engineering Southern Medical University Guangzhou China

**Keywords:** CXCR4, JAK/STAT, renal fibrosis, tubular cell, β‐catenin

## Abstract

Chronic kidney disease (CKD) has a high prevalence worldwide. Renal fibrosis is the common pathological feature in various types of CKD. However, the underlying mechanisms are not determined. Here, we adopted different CKD mouse models and cultured human proximal tubular cell line (HKC‐8) to examine the expression of C‐X‐C motif chemokine receptor 4 (CXCR4) and β‐catenin signalling, as well as their relationship in renal fibrosis. In CKD mice and humans with a variety of nephropathies, CXCR4 was dramatically up‐regulated in tubules, with a concomitant activation of β‐catenin. CXCR4 expression level was positively correlated with the expression of β‐catenin target MMP‐7. AMD3100, a CXCR4 receptor blocker, and gene knockdown of CXCR4 significantly inhibited the activation of JAK/STAT and β‐catenin signalling, protected against tubular injury and renal fibrosis. CXCR4‐induced renal fibrosis was inhibited by treatment with ICG‐001, an inhibitor of β‐catenin signalling. In HKC‐8 cells, overexpression of CXCR4 induced activation of β‐catenin and deteriorated cell injury. These effects were inhibited by ICG‐001. Stromal cell–derived factor (SDF)‐1α, the ligand of CXCR4, stimulated the activation of JAK2/STAT3 and JAK3/STAT6 signalling in HKC‐8 cells. Overexpression of STAT3 or STAT6 decreased the abundance of GSK3β mRNA. Silencing of STAT3 or STAT6 significantly blocked SDF‐1α‐induced activation of β‐catenin and fibrotic lesions. These results uncover a novel mechanistic linkage between CXCR4 and β‐catenin activation in renal fibrosis in association with JAK/STAT/GSK3β pathway. Our studies also suggest that targeted inhibition of CXCR4 may provide better therapeutic effects on renal fibrosis by inhibiting multiple downstream signalling cascades.

## 1 INTRODUCTION

CKD has a high prevalence in worldwide population.^1^ Even though with multiple pathological changes, various types of CKD are characterized with the common pathological feature of renal fibrosis. However, to date, there are not completely effective therapeutics to renal fibrosis.^2^ Although dialysis and transplantation serve as the alternative treatments to renal fibrosis, they could only provide very limited remission for the frequent occurrence of cardiovascular complications in dialysis patients and high rate of recurrence of the original diseases in the transplanted kidneys.^3^, ^4^ Hence, to better understand the underlying mechanisms and identify therapeutic targets of renal fibrosis would be of great importance to CKD.

As the most abundant cells in kidneys, renal tubular cells play fundamental roles in executing renal functions.^5^ Upon damage, they could undergo apoptosis, epithelial‐mesenchymal transition, cellular senescence and dedifferentiation. Large bodies of evidences have reported that damaged tubular cells could secret transforming growth factor‐β1 (TGF‐β1), the critical mediator leading to interstitial fibroblasts activation. However, the underlying mechanisms of renal tubular cell injury need to be elucidated.

As a G‐protein–coupled seven‐span transmembrane receptor, C‐X‐C motif chemokine receptor 4 (CXCR4) serves as the key molecule to leash haematopoietic stem cells to quiescence in bone marrow.^6^ Recently, some reports indicate CXCR4 plays a crucial role in various types of CKD such as rapidly progressive glomerulonephritis (RPGN), IgA nephropathy (IgAN), lupus and tubulo‐interstitial nephritis.^7^, ^8^, ^9^, ^10^, ^11^ Although CXCR4 exists in various types of cells such as podocytes, macrophages and endothelial cells, it is predominantly localized in tubular cells, especially proximal tubular epithelial cells.^7^, ^8^, ^10^, ^12^ Through the binding of CXC chemokine ligand 12 (CXCL12; stromal cell–derived factor 1 (SDF‐1)), CXCR4 exerts multiple effects in cell survival, differentiation and injury.^6^, ^7^, ^13^ However, the role of CXCR4 in renal tubular cell injury and the underlying mechanisms remain poorly understood.

Wnt/β‐catenin signalling is a conserved developmental pathway that plays a critical role in organ development.^14^ Different from its silent expression in normal adult's kidneys, Wnt/β‐catenin signalling is dramatically up‐regulated in CKD‐affected kidneys. The activation of Wnt/β‐catenin signalling is highly associated with oxidative stress, inflammation and cellular senescence.^15^, ^16^, ^17^, ^18^ Upon the binding of Wnt ligands to the receptors frizzled (Fzd) and lipoprotein receptor–related protein (LRP) 5/6, GSK3β activity is repressed, and then, β‐catenin would be released and activated to trigger cell injury and renal fibrosis. Although β‐catenin could be up‐regulated in multiple cells such as podocytes, interstitial fibroblasts, endothelial cells and inflammatory cells,^15^, ^17^, ^19^, ^20^, ^21^ it is predominantly localized in renal tubular cells in injured kidneys.^18^, ^22^ These observations suggest the potential relationship between CXCR4 and β‐catenin in renal tubular cell injury and fibrosis.

In this study, we examined CXCR4 and β‐catenin signalling and assessed their relationship in vivo and in vitro. The results indicate CXCR4 plays a crucial role in mediating renal tubular cell injury and is associated with activation of β‐catenin. Furthermore, JAK/STAT/GSK3β pathway mediates SDF‐1α/CXCR4‐induced activation of β‐catenin. These findings suggest that CXCR4 mediates renal fibrosis through activating JAK/STAT/GSK3β/β‐catenin pathway.

## 2 MATERIALS AND METHODS

### 2.1 Animal models

Male C57BL/6 mice, weighing 22‐24 g, were purchased from Southern Medical University Animal Center (Guangzhou, China) and housed in a standard environment on a regular light/dark cycle with free access to water and chow. For the unilateral ureteral obstruction (UUO) model, male C57BL/6 mice were performed double‐ligating of the left ureter using 4‐0 silk after a midline abdomen incision. Sham‐operated mice had their ureters exposed and manipulated but not ligated. Mice were killed 7 and 14 days after UUO. The ischaemia‐reperfusion injury (IRI) model was established by bilateral renal pedicles clipped for 32 minutes using microaneurysm clamps. During the ischaemic period, body temperature was maintained between 37°C and 38°C using a temperature‐controlled heating system. After removal of the clamps, reperfusion of the kidneys was visually confirmed. Mice were killed 7 and 10 days after IRI. For the unilateral IRI (UIRI) model, male C57BL/6 mice were subjected to unilateral renal IRI by an established protocol, as described previously.^18^ Briefly, left renal pedicles were clipped for 35 minutes using microaneurysm clamps for IRI injury. After removal of the clamps, reperfusion of the kidneys was visually confirmed. Ten days later, the intact right kidney was removed via a right flank incision. The mice were killed 11 days after IRI surgery. AMD3100 (A5602; Sigma‐Aldrich, St. Louis, MO) and ICG‐001(847591‐62‐2, Chemleader, Shanghai, China) were intraperitoneally injected at the doses of 1 mg/kg/d and 5 mg/kg/d, respectively. The detailed experimental designs were presented in Figures 2A, 4A, 6A and 10A. Mouse CXCR4siRNA sequence (5’‐CGAUCAGUGUGAGUAUAUATT‐3’) was ligated into an shRNA expression plasmid (pLVX‐shRNA). Groups of mice were injected with the mouse CXCR4 expression plasmid (pFlag‐CXCR4) or shRNA expression plasmid (pLVX‐shCXCR4) by rapid injection of a large volume of DNA solution through the tail vein, as described previously.^23^

### 2.2 Cell culture and treatment

Human proximal tubular epithelial cells (HKC‐8) were provided by Dr L. Racusen (Johns Hopkins University, Baltimore, MD). Cell culture was carried out according to the procedures described previously.^23^ HKC‐8 cells were treated with SDF‐1α (SRP4388; Sigma‐Aldrich, St. Louis, MO), Wnt3a (H17001; Sigma‐Aldrich, St. Louis, MO) or ICG‐001 (847591‐62‐2, Chemleader, Shanghai, China) at indicated concentrations. Some cells were transfected with the CXCR4 expression plasmid (pFlag‐CXCR4), STAT3 expression plasmid (pFlag‐STAT3) or STAT6 expression plasmid (pHA‐STAT6). Oligonucleotide siRNA duplex was synthesized by Shanghai Gene Pharma (Shanghai, China). Human STAT3 siRNA sequence was 5’‐ GCAACAGAUUGCCUGCAUUTT‐3’, and STAT6 siRNA was 5’‐ CCAAGACAACAAUGCCAAATT‐3’. The sequence of scramble siRNA was 5’‐UUCUCCGAACGUGUCACGUTT‐3’. The transfection of expression plasmid or siRNA in HKC‐8 cells was carried out with lipofectamine 2000 (Invitrogen, Carlsbad, CA) according to the manufacturer's instruction.

### 2.3 Western blot analysis

Protein expression levels were analysed by Western blot analysis as described previously.^15^, ^18^ The primary antibodies used were as follows: anti‐SDF‐1α (ab25117; Abcam), anti‐fibronectin (F3648; Sigma‐Aldrich), anti‐α‐SMA (ab5694; Abcam), anti‐collagen I (ab34710; Abcam), anti‐vimentin (SAB1305447, Sigma‐Aldrich), anti‐CXCR4 (BA0747‐2; Boster), anti‐p‐GSK3β (ser 9) (5558; Cell Signaling Technology), anti‐β‐catenin (610 154; BD Transduction Laboratories), anti‐PAI‐1 (AF3828; R&D Systems), anti‐snail1 (ab180714; Abcam), anti‐MMP‐7 (104 658; GTX), anti‐E‐cadherin (3195; Cell Signaling Technology), anti‐active β‐catenin (19807s; Cell Signaling Technology), anti‐Kim1 (BA3537; Boster, Wuhan, China), anti‐α‐tubulin (RM2007, Ray Antibody Biotech, Beijing, China) and p‐STAT antibody sampler kit (9914; Cell Signaling Technology), p‐JAK family antibody sampler kit (97 999; Cell Signaling Technology), STAT antibody sampler kit (9939; Cell Signaling Technology) and anti‐GAPDH (RM2000, Ray Antibody Biotech, Beijing, China).

### 2.4 Histology and immunohistochemical staining

Paraffin‐embedded mouse kidney sections (4 µm thickness) were prepared by a routine procedure. Periodic acid‐Schiff (PAS), Masson trichrome staining and Sirius red staining (DC0040, Leagene Biotechnology, Beijing, China) were performed by a standard protocol or according to the manufacturer's instruction. Immunohistochemical staining was performed using routine protocol. Primary antibodies used were as follows: CXCR4 (BA0747‐2; Boster), β‐catenin (610 154; BD Transduction Laboratories), collagen I (ab34710; Abcam), α‐SMA (ab5694; Abcam), E‐cadherin (3195; Cell Signaling Technology), fibronectin (F3648; Sigma‐Aldrich) and active β‐catenin (19807s; Cell Signaling Technology), anti‐p‐GSK3β (ser 9) (5558; Cell Signaling Technology), p‐STAT3 or p‐STAT6 (9914; Cell Signaling Technology) and p‐JAK2 (97 999; Cell Signaling Technology).

### 2.5 Immunofluorescence staining and confocal microscopy

Frozen kidney sections (3 µm) were fixed with 4% paraformalin for 15 minutes at room temperature. HKC‐8 cells cultured on coverslips were fixed with cold methanol:acetone (1:1) for 10 minutes at −20°C. The slides were blocked with normal donkey serum and incubated with primary antibodies as follows: anti‐fibronectin (F3648, Sigma‐Aldrich), anti‐β‐catenin antibody (ab15180; Abcam), anti‐STAT3 antibody (4904; Cell Signaling Technology), anti‐STAT6 antibody (5397; Cell Signaling Technology) and anti‐E‐cadherin (3195; Cell Signaling Technology). After washing, the slides were incubated with Cy3‐ or Cy2‐conjugated donkey anti‐mouse or donkey anti‐rabbit IgG (Jackson Immuno‐Research Laboratories, West Grove, PA). Nuclei were stained with DAPI (Sigma‐Aldrich) according to the manufacturer's instruction. Images were taken by confocal microscopy (Leica TCS SP2 AOBS; Leica Microsystems, Buffalo Grove, IL) or Olympus DP80 microscope with EMCCD camera.

### 2.6 Reverse transcriptase (RT)‐PCR

Total RNA was prepared using TRIzol RNA isolation system (Life Technologies, Grand Island, NY) according to the manufacturer's instruction. The first strand of complementary DNA was synthesized using 1 μg of RNA in 20 μL of reaction buffer using AMV‐RT and random primers at 42°C for 60 minutes. PCR amplification was performed using a HotStarTaq Master Mix kit (Qiagen, Valencia, CA). The sequences of the primer pairs are shown in Table S1.

### 2.7 Serum creatinine (Scr) and blood urea nitrogen (BUN) assay

Determination of Scr and BUN was analysed by an automatic chemistry analyzer (AU480; Beckman Coulter Inc; Kraemer Boulevard Brea, CA). The level of Scr or BUN was expressed as mg/dL or mmol/L, respectively.

### 2.8 Statistical analyses

All data examined were expressed as mean ± SEM. Statistical analysis of the data was carried out using SPSS 19.0 (SPSS Inc, Chicago, IL). Comparison between groups was made using one‐way ANOVA followed by Student‐Newman‐Keuls test or Dunnett's T3 procedure. *P* < .05 was considered significant.

## 3 RESULTS

### 3.1 CXCR4 is up‐regulated in fibrotic kidneys and accompanied by activation of β‐catenin

**Figure 1:**
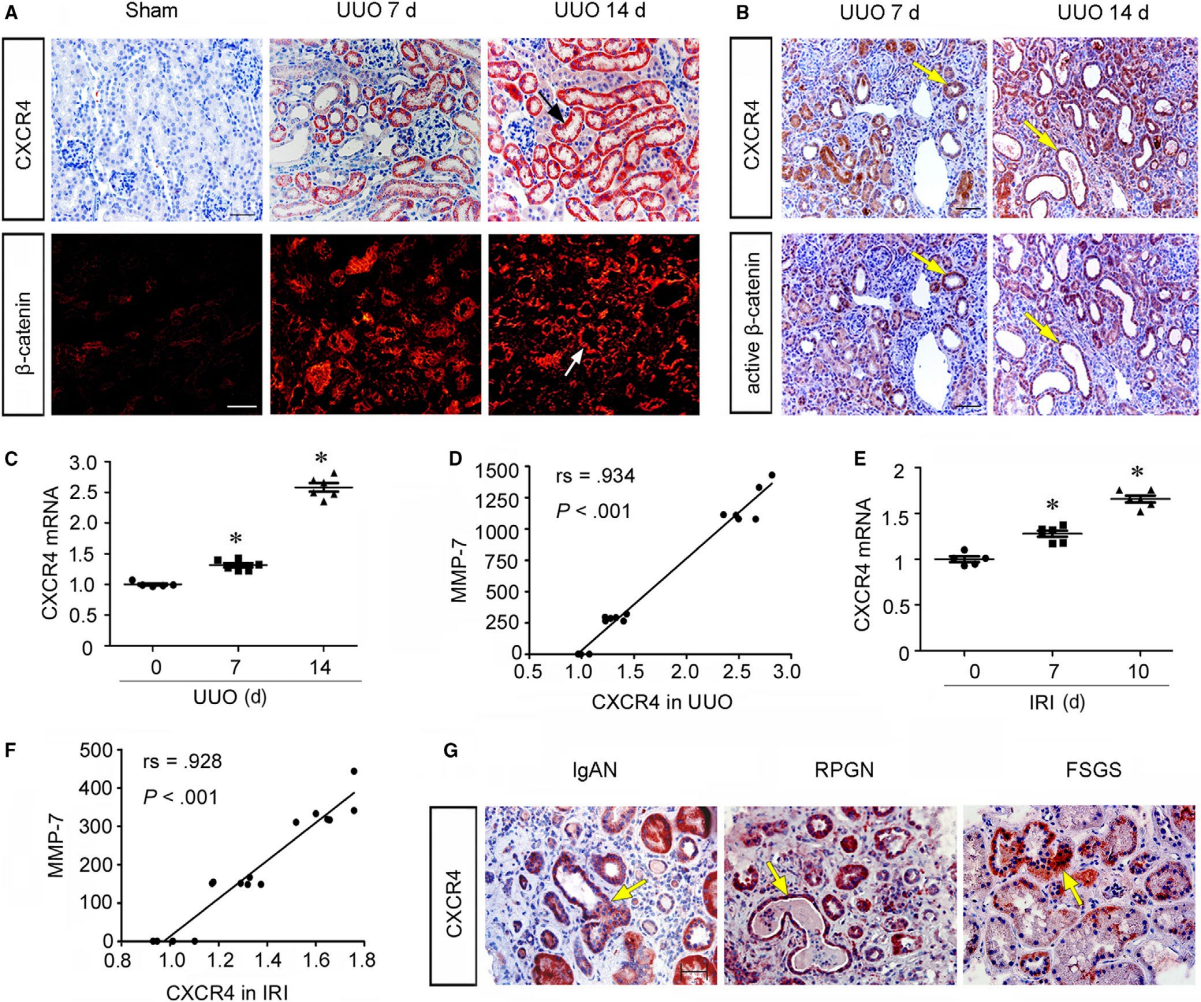
CXCR4 increases in fibrotic kidneys and is associated with activation of β‐catenin signalling. A, Representative micrographs show the expression of CXCR4 was up‐regulated in unilateral ureteral obstruction (UUO) mice. Paraffin kidney sections were stained with an antibody against CXCR4. Frozen sections were stained with an antibody against β‐catenin. Arrows indicate positive staining. Scale bar, 50 μm. B, Sequential paraffin‐embedded kidney sections from UUO mice were immunostained for CXCR4 and active β‐catenin. Colocalization of CXCR4 and active β‐catenin in renal tubules 7 or 14 d after UUO were indicated by yellow arrows. Scale bar, 50 μm. C, The relative abundance of CXCR4 mRNA was analysed by quantitative real‐time PCR in UUO mice. *P < .05 versus sham controls (n = 5‐6). D, Linear regression analysis showing an positive correlation between renal CXCR4 and MMP‐7 mRNA. The correlation coefficient (rs) is shown. E, The relative abundance of CXCR4 mRNA was analysed by quantitative real‐time PCR in IRI mice. *P < .05 versus sham controls (n = 5‐6). F, Linear regression analysis showing an positive correlation between renal CXCR4 and MMP‐7 mRNA in IRI mice. The correlation coefficient (rs) is shown. G, Representative micrographs show CXCR4 was expressed predominantly in renal tubular cells in humans with IgAN, RPGN and FSGS. Yellow arrows indicate positive staining in tubules. Scale bar, 50 μm. CXCR4, C‐X‐C chemokine receptor type 4; FSGS, focal segmental glomerulosclerosis; IgAN, immunoglobulin A nephropathy; RPGN, rapidly progressive glomerulonephritis.

To identify the role of CXCR4 in renal fibrosis, we first examined the expression of CXCR4 and CXCL12 (SDF‐1α), the ligand of CXCR4, in various CKD models. The protein expression of CXCL12 (SDF‐1α) was elevated time‐dependently in UUO and IRI mice (Figure S1). Consistently, as shown in Figure 1A, the expression of CXCR4 was time‐dependently up‐regulated in UUO mice and predominantly localized in renal tubular cells. Furthermore, the expression of β‐catenin was also evidently up‐regulated in UUO‐affected kidneys, especially at the late stage of UUO. Notably, both CXCR4 and β‐catenin expression were up‐regulated in renal tubular cells. To clarify the correlation between CXCR4 and β‐catenin, we examined the colocalization of CXCR4 and active β‐catenin in sequential paraffin‐embedded kidney sections. As shown in Figure 1B, the expression of CXCR4 largely colocalized with active β‐catenin in tubular cells. We then analysed mRNA expression levels of CXCR4 and MMP‐7, an important downstream target of β‐catenin.^24^ As shown in Figure 1C and Figure S2, CXCR4 and MMP‐7 mRNA levels were significantly up‐regulated in UUO mice in a time‐dependent fashion. Notably, there was a significant positive correlation between CXCR4 and MMP‐7 (Figure 1D). Similarly, in IRI kidneys, CXCR4 and MMP‐7 mRNA abundance also increased and positively correlated (Figure 1E and F, Figure S2). It is notable that the increased abundance of CXCR4 mRNA was much less than that of MMP‐7, suggesting the amplification in signalling cascades. We next investigated CXCR4 expression in humans with immunoglobulin A nephropathy (IgAN), rapidly progressive glomerulonephritis (RPGN) and focal segmental glomerulosclerosis (FSGS). A shown in Figure 1G, CXCR4 was predominantly localized in renal tubular cells in human diseased kidneys, suggesting the intimate correlation of CXCR4 in tubular cell injury and renal fibrosis.

### 3.2 Blockade of CXCR4 ameliorates renal fibrosis in UUO mice

**Figure 2:**
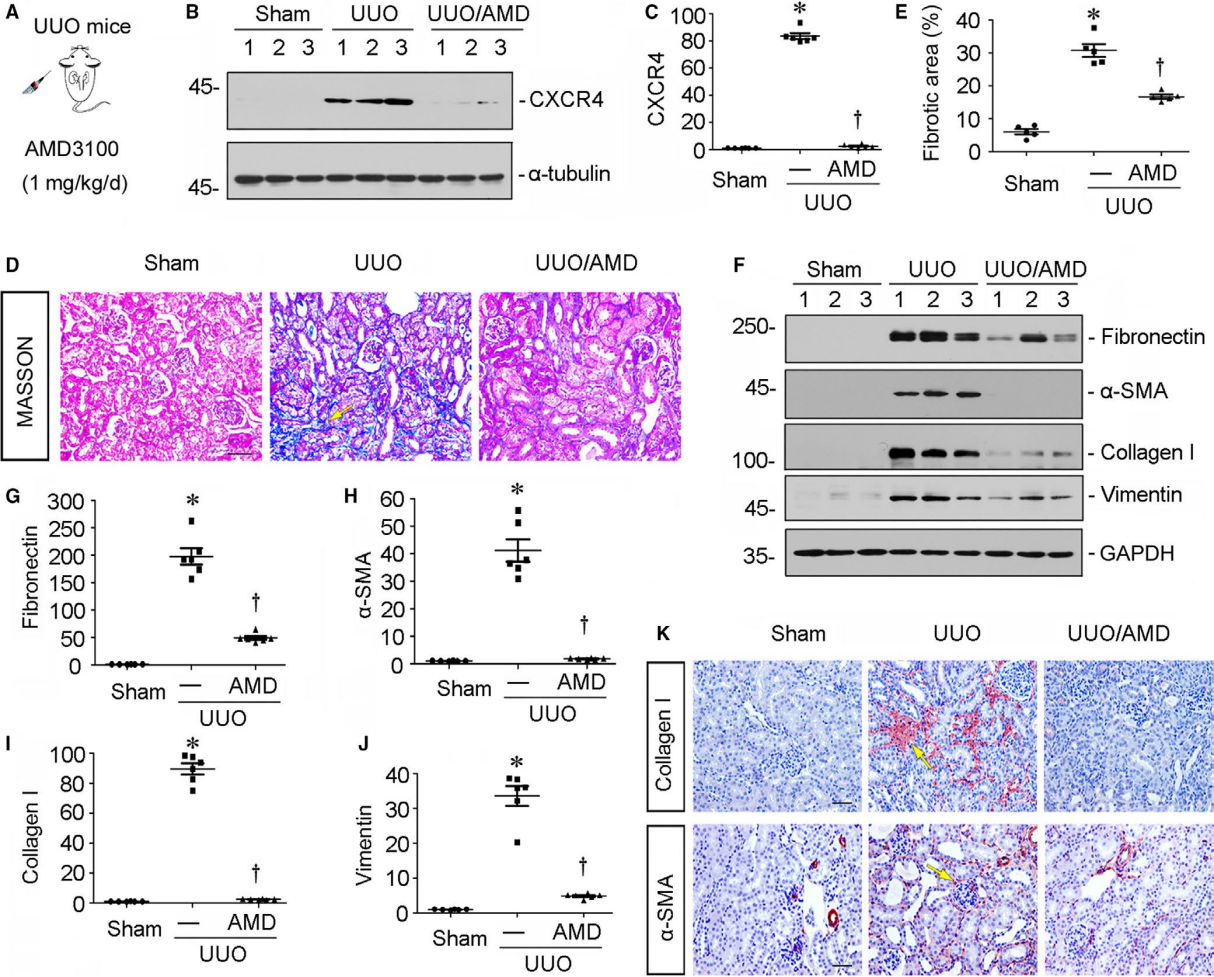
Blockade of CXCR4 ameliorates renal fibrosis in UUO mice. A, Experimental design. AMD3100 was dissolved in sterile saline and intraperitoneally injected at the dose of 1 mg/kg/d after UUO operation. The mice were killed 7 d after UUO surgery. B‐C, Representative (B) Western blots and graphical representation of (C) CXCR4 protein expression in different groups. *P < .05 versus sham control mice (n = 5‐6); †*P* < .05 versus UUO mice (n = 5‐6). D, Representative micrographs show renal fibrotic lesions in different groups. Paraffin kidney sections were performed Masson trichrome staining. Yellow arrow indicates positive staining. Scale bar, 50 μm. E, Graphical representation of the quantification of renal fibrotic lesions. *P < .05 versus sham control mice (n = 5); †*P* < .05 versus UUO mice (n = 5‐6). F‐J, Representative (F) Western blots and graphical representations of (G) fibronectin, (H) α‐SMA, (I) collagen I and (J) vimentin in three groups. Numbers 1‐3 indicate each individual animal in given group. *P < .05 versus sham control mice (n = 5‐6); †*P* < .05 versus UUO mice (n = 5‐6). K, Representative micrographs show collagen I and α‐SMA expression in three groups. Paraffin kidney sections were immunostained with antibodies against collagen I and α‐SMA. Yellow arrows indicate positive staining. Scale bar, 50 μm

To investigate the potential role of CXCR4 in renal fibrosis, we injected mice with AMD3100,^7^ a specific inhibitor of CXCR4 signalling in UUO model (Figure 2A). The mice were killed 7 days after UUO surgery. The expression of CXCR4 was first analysed. As shown in Figure 2B and C, the protein expression of CXCR4 was significantly up‐regulated in UUO mice. However, treatment with AMD3100 obviously inhibited the expression of CXCR4. We then performed Masson trichrome staining and the quantification of renal fibrotic lesions. As shown in Figure 2D and E, treatment with AMD3100 significantly retarded renal fibrosis in UUO mice. Similar results were observed when the protein expression levels of fibronectin, α‐SMA, collagen I and vimentin were assessed by Western blot analyses (Figure 2F‐J). Furthermore, the immunostaining results of collagen I and α‐SMA also confirmed the therapeutic effects of AMD3100 on matrix deposition in UUO mice (Figure 2K).

### 3.3 Blockade of CXCR4 represses β‐catenin activation in UUO mice

**Figure 3:**
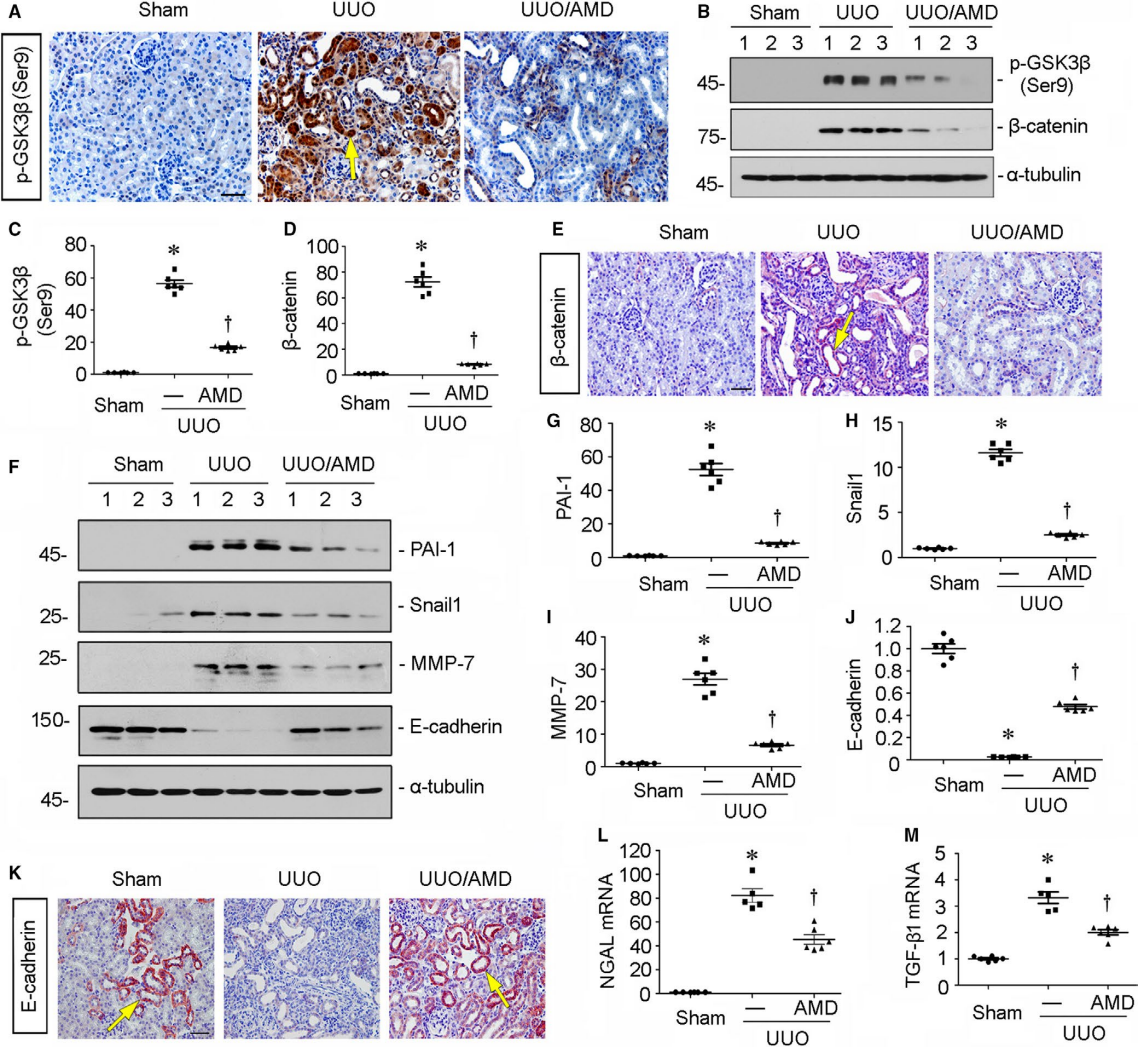
Blockade of CXCR4 represses β‐catenin signalling in UUO mice. A, Representative micrographs show the expression of p‐GSK3β (Ser9) in different groups. Paraffin kidney sections were immunostained with an antibody against p‐GSK3β (Ser9).Yellow arrow indicates positive staining. Scale bar, 50 μm. B‐D, Representative (B) Western blots and graphical representation of (C) p‐GSK3β (Ser9) and (D) β‐catenin in three groups as indicated. Numbers 1‐3 indicate each individual animal in given groups. *P < .05 versus sham control mice (n = 5‐6); †*P* < .05 versus UUO mice (n = 5‐6). E, Representative micrographs show renal expression of β‐catenin in different groups. Paraffin kidney sections were immunostained with an antibody against β‐catenin. Yellow arrow indicates positive staining. Scale bar, 50 μm. F‐J, Representative (F) Western blots and graphical representations of (G) PAI‐1, (H) Snail 1, (I) MMP‐7 and (J) E‐cadherin protein expression in three groups. Numbers 1‐3 indicate each individual animal in a given group. *P < .05 versus sham control mice (n = 5‐6); †*P* < .05 versus UUO mice (n = 5‐6). K, Representative micrographs show renal expression of E‐cadherin. Paraffin kidney sections were immunostained with an antibody against E‐cadherin. Yellow arrows indicate positive staining. Scale bar, 50 μm. L and M, Graphical representations show the relative abundance of (L) neutrophil gelatinase associated lipocalin (NGAL) and (M) transforming growth factor‐β1 (TGF‐β1) mRNA in three groups. *P < .05 versus sham control mice (n = 5‐6); †*P* < .05 versus UUO mice (n = 5‐6)

We then examined the correlation between CXCR4 and β‐catenin. First, we assessed the expression of GSK3β, a serine/threonine protein kinase that mediates β‐catenin degradation and its phosphorylation at Ser9 suggests the deactivation of GSK3β.^25^ As shown in Figure 3A, B and C, the protein expression of p‐GSK3β (Ser9) was extremely up‐regulated in UUO mice, but blocked by administration of AMD3100, the antagonist of CXCR4 receptor.^7^ Similar results were observed when the expression of β‐catenin was analysed by Western blot (Figure 3B and D) and immunohistochemical staining (Figure 3E). We next checked renal expression of plasminogen activator inhibitor‐1 (PAI‐1), Snail 1 and matrix metalloproteinase‐7 (MMP‐7), the three downstream targets of β‐catenin and the important players in tubular epithelial‐mesenchymal transition and fibrotic injury.^26^, ^27^ As shown in Figure 3F‐I, all of them were significantly up‐regulated in UUO mice, but inhibited by administration of AMD3100. Moreover, the expression of E‐cadherin, an important protein maintaining normal epithelial integrity,^28^ was greatly down‐regulated in UUO‐affected kidneys. However, it was largely preserved by treatment with AMD3100 (Figure 3F, J, and K). To further confirm the role of CXCR4 in tubular cell injury, we examined the mRNA expression of neutrophil gelatinase–associated lipocalin (NGAL) and transforming growth factor‐β1 (TGF‐β1), two markers of tubular cell injury^29^, ^30^ by quantitative real‐time PCR. As shown in Figure 3L and M, administration of AMD3100 significantly blocked UUO‐induced up‐regulation of NGAL and TGF‐β1. It was previously reported that signalling crosstalk between β‐catenin and TGF‐β1 is an important mechanism in tubular epithelial cell injury.^31^ Hence, these data further suggest that CXCR4 plays an important role in tubular cell injury and is associated with β‐catenin activation.

### 3.4 Blockade of CXCR4 mitigates renal fibrosis in UIRI mice

**Figure 4:**
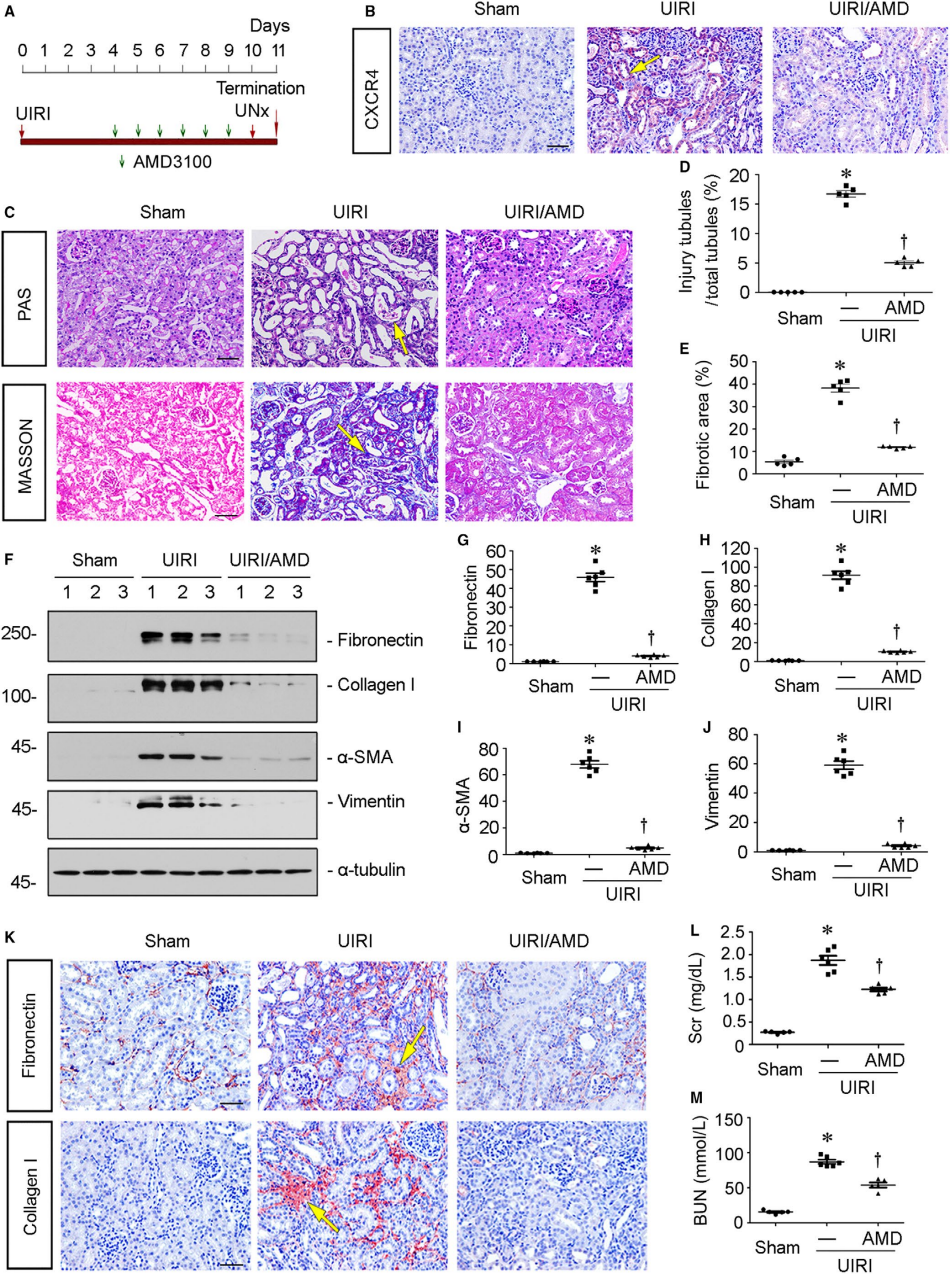
Blockade of CXCR4 mitigates renal fibrosis in UIRI mice. A, Experimental design. Green arrows indicate the injections of AMD3100. Red arrows indicate the timing of renal IRI surgery. B, Representative micrographs show renal expression of CXCR4 in different groups. Paraffin kidney sections were immunostained with an antibody against CXCR4. Arrow indicates positive staining. Scale bar, 50 μm. C, Representative micrographs show PAS and Masson trichrome staining in different groups. Arrows indicate positive staining. Scale bar, 50 μm. D and E, Graphical representations of quantitative analyses of (D) injured tubules and (E) renal fibrotic lesions in three groups. Kidney sections were subjected to PAS and Masson trichrome staining. At least 20 randomly selected fields were evaluated under x400 magnification, and results were averaged for each kidney. *P < .05 versus sham control. †*P* < .05 versus UIRI alone (n = 5). F‐J, Representative (F) Western blots and graphical representations of (G) fibronectin, (H) collagen I, (I) α‐SMA and (J) vimentin protein expression levels in three groups. Numbers 1‐3 indicate each individual animal in given group. *P < .05 versus sham control (n = 5‐6); †*P* < .05 versus UIRI alone (n = 5‐6). Scale bar, 50 μm. (K)Representative micrographs show renal expression of fibronectin and collagen I in three groups. Paraffin kidney sections were immunostained with antibodies against fibronectin and collagen I. Yellow arrows indicate positive staining. Scale bar, 50 μm. L and M, Quantitative analysis of (L) serum creatinine (Scr) and (M) blood urea nitrogen (BUN) levels in three groups as indicated. *P < .05 versus sham control (n = 5‐6); †*P* < .05 versus UIRI alone (n = 5‐6). Scr was expressed as milligrams per decilitre, and BUN was expressed as millimole per litre

To further confirm the role of CXCR4 in renal fibrotic lesions, we carried out the mouse model of unilateral ischaemia‐reperfusion injury (UIRI). Groups of mice were subjected to UIRI and administered AMD3100 at the dose of 1 mg/kg/d (Figure 4A). The immunostaining results revealed that the up‐regulation of CXCR4 in UIRI mice was blocked by administration of AMD3100 (Figure 4B). Furthermore, PAS staining showed tubular cell injury, characterized by tubular dilation, hyaline casts and tubular atrophy with thickened basement membranes, as well as detached epithelial cells in tubular lumens, was dramatically induced in UIRI mice. However, these effects were clearly blocked by administration of AMD3100 (Figure 4C). Similarly, Masson trichrome staining showed AMD3100 largely decreased the fibrotic lesions in UIRI mice (Figure 4C).The extent levels of tubular cell injury and fibrotic lesions were assessed by a semiquantitative analysis and presented in Figure 4D and E. Consistently, the protein expression levels of fibronectin, collagen I, α‐SMA and vimentin were tested by Western blot analyses. As shown in Figure 4F‐J, all of them were significantly inhibited by administration of AMD3100. Similar results were observed when fibronectin and α‐SMA were analysed by immunostaining (Figure 4K). We then tested the levels of serum creatinine (Scr) and blood urea nitrogen (BUN). As shown in Figure 4L and M, treatment with AMD3100 significantly reduced their up‐regulation in UIRI mice. These data further indicate that CXCR4 activation serves as a major driver of tubular cell injury and renal fibrosis.

### 3.5 Blockade of CXCR4 represses β‐catenin signalling in UIRI mice

**Figure 5:**
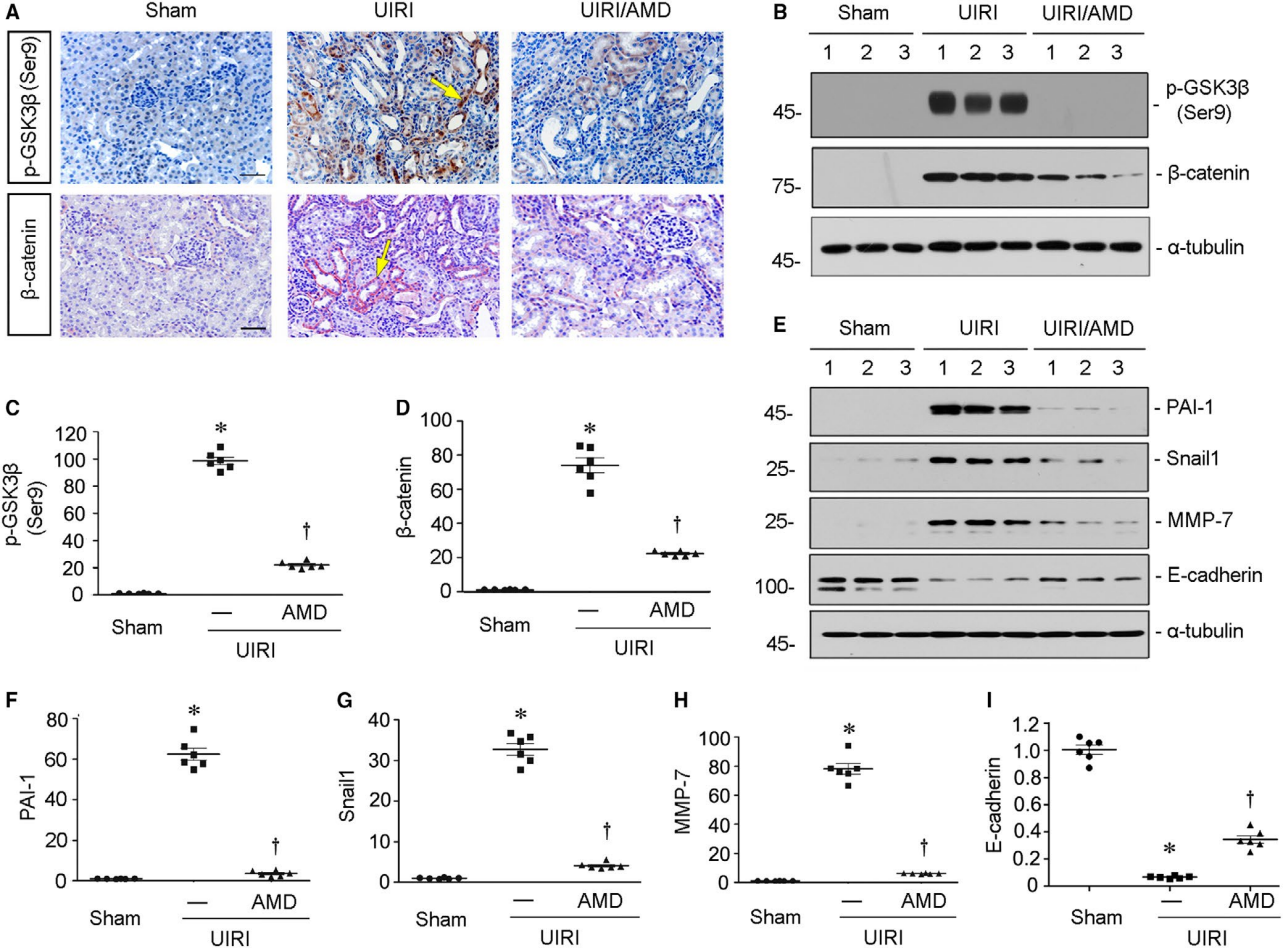
Blockade of CXCR4 represses β‐catenin signalling in UIRI mice. A, Representative micrographs show renal expression of p‐GSK3β (Ser9) and β‐catenin in three groups. Paraffin sections were immunostained with antibodies against p‐GSK3β (Ser9) and β‐catenin. Yellow arrows indicate positive staining. Scale bar, 50 μm. B‐D, Representative (B) Western blots and graphical representations of (C) p‐GSK3β (Ser9) and (D) β‐catenin in three groups. Numbers 1‐3 indicate each individual animal in a given group. *P < .05 versus sham control (n = 5‐6); †*P* < .05 versus UIRI alone (n = 5‐6). E‐I, Representative (E) Western blots and graphical representations of (F) PAI‐1, (G) Snail 1, (H) MMP‐7 and (I) E‐cadherin in three different groups. Numbers 1‐3 indicate each individual animal in a given group. *P < .05 versus sham controls (n = 5‐6); †*P* < .05 versus UIRI alone (n = 5‐6)

We then assessed β‐catenin signalling in UIRI mice. First, the expression of p‐GSK3β (Ser9) was assessed. As shown in Figure 5A, B and C, administration of AMD3100 significantly inhibited the up‐regulation of p‐GSK3β (Ser9) in UIRI mice. Consequently, the expression of β‐catenin was induced by UIRI injury but inhibited by treatment with AMD3100 (Figure 5A, B and D), suggesting the role of CXCR4 in β‐catenin activation. Similarly, the protein expression levels of PAI‐1, Snail 1 and MMP‐7, the downstream targets of β‐catenin, were significantly down‐regulated by treatment with AMD3100 in UIRI mice (Figure 5E‐H). We next analysed E‐cadherin, the tubular epithelial cell marker. As shown in Figure 5E and I, the down‐regulation of E‐cadherin induced by UIRI injury was significantly reversed by administration of AMD3100.

### 3.6 Inhibition of β‐catenin retards CXCR4‐aggravated renal fibrosis in UUO model

**Figure 6:**
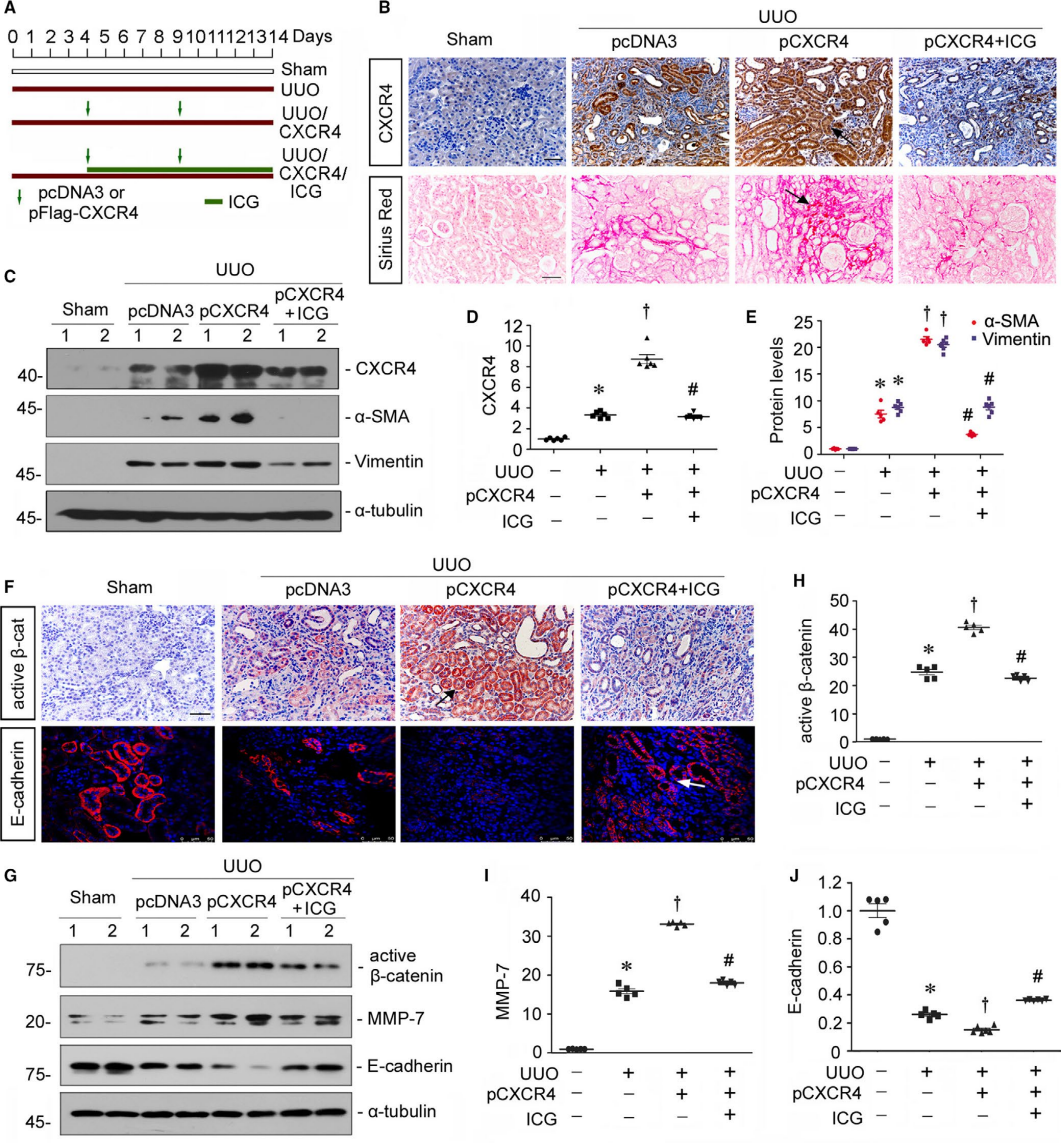
ICG‐001 inhibits CXCR4‐aggravated renal fibrosis in UUO model. A, Experimental design. Green arrows indicate the injections of empty vector (pcDNA3) or a Flag‐tagged CXCR4 expression plasmid (pFlag‐CXCR4). Green bar indicates the timing of administration of ICG‐001. B, Representative micrographs show the expression of CXCR4 and Sirius red staining in four groups. Paraffin kidney sections were immunostained with an antibody against CXCR4 or performed Sirius red staining. Black arrows indicate positive staining. Scale bar, 50 μm. C‐E, Representative (C) Western blots and graphical representations of (D) CXCR4, (E) α‐SMA and vimentin in four groups. Numbers 1‐2 represent individual animal in a given group. *P < .05 versus sham controls (n = 5‐6); †*P* < .05 versus UUO alone (n = 5‐6); #*P* < .05 versus UUO plus pCXCR4 (pFlag‐CXCR4) (n = 5‐6). F, Representative micrographs show renal expression of active β‐cat (active β‐catenin) and E‐cadherin in four groups. Paraffin kidney sections were immunostained with an antibody against active β‐catenin or E‐cadherin. Arrows indicate positive staining. Scale bar, 50 μm. G‐J, Representative (G) Western blots and graphical representations of (H) active β‐catenin, (I) MMP‐7 and (J) E‐cadherin in four groups. *P < .05 versus sham controls (n = 5‐6); †*P* < .05 versus UUO alone (n = 5‐6); #*P* < .05 versus UUO plus pCXCR4 (pFlag‐CXCR4) (n = 5‐6)

To further establish the role of CXCR4 in β‐catenin signalling, mice were delivered a flag‐tagged CXCR4 expression plasmid (pFlag‐CXCR4) by a hydrodynamic‐based gene therapy^23^ and administered ICG‐001, a small molecule inhibiting β‐catenin‐mediated gene transcription in a CBP‐dependent manner,^22^ after UUO surgery (Figure 6A).The expression of CXCR4 was identified by immunohistochemistry and Western blot analyses. As shown in Figure 6B‐D, CXCR4 protein level was up‐regulated in UUO mice, predominantly in renal tubular cells, but further elevated by injection of CXCR4 plasmid. However, administration of ICG‐001 could greatly inhibit it. We next investigated renal fibrotic lesions through Sirius red staining, a technique that collagen fibre would be stained as red colour. As shown in Figure 6B, ectopic expression of CXCR4 evidently aggravated renal fibrosis in UUO mice. However, administration of ICG‐001 largely retarded CXCR4‐induced renal fibrotic lesions. Similar results were observed when the protein expression levels of α‐SMA and vimentin were assessed by Western blot analyses (Figure 6C and E). We next assessed β‐catenin signalling. As shown in Figure 6F, the expression of active β‐catenin was significantly induced by ectopic expression of CXCR4 in UUO mice, but diminished by administration of ICG‐001. Similar results were observed when the protein expression level of active β‐catenin was analysed by Western blot analyses (Figure 6G and H). Furthermore, the protein expression level of MMP‐7 was also significantly inhibited by treatment with ICG‐001 (Figure 6G and I). Moreover, administration of ICG‐001 significantly reversed CXCR4‐aggravated down‐regulation of E‐cadherin in UUO mice (Figure 6F, G and J). These data further suggest the mediating role of β‐catenin in CXCR4‐induced renal fibrosis.

### 3.7 CXCR4/β‐catenin axis plays a critical role in cell injury in vitro

**Figure 7:**
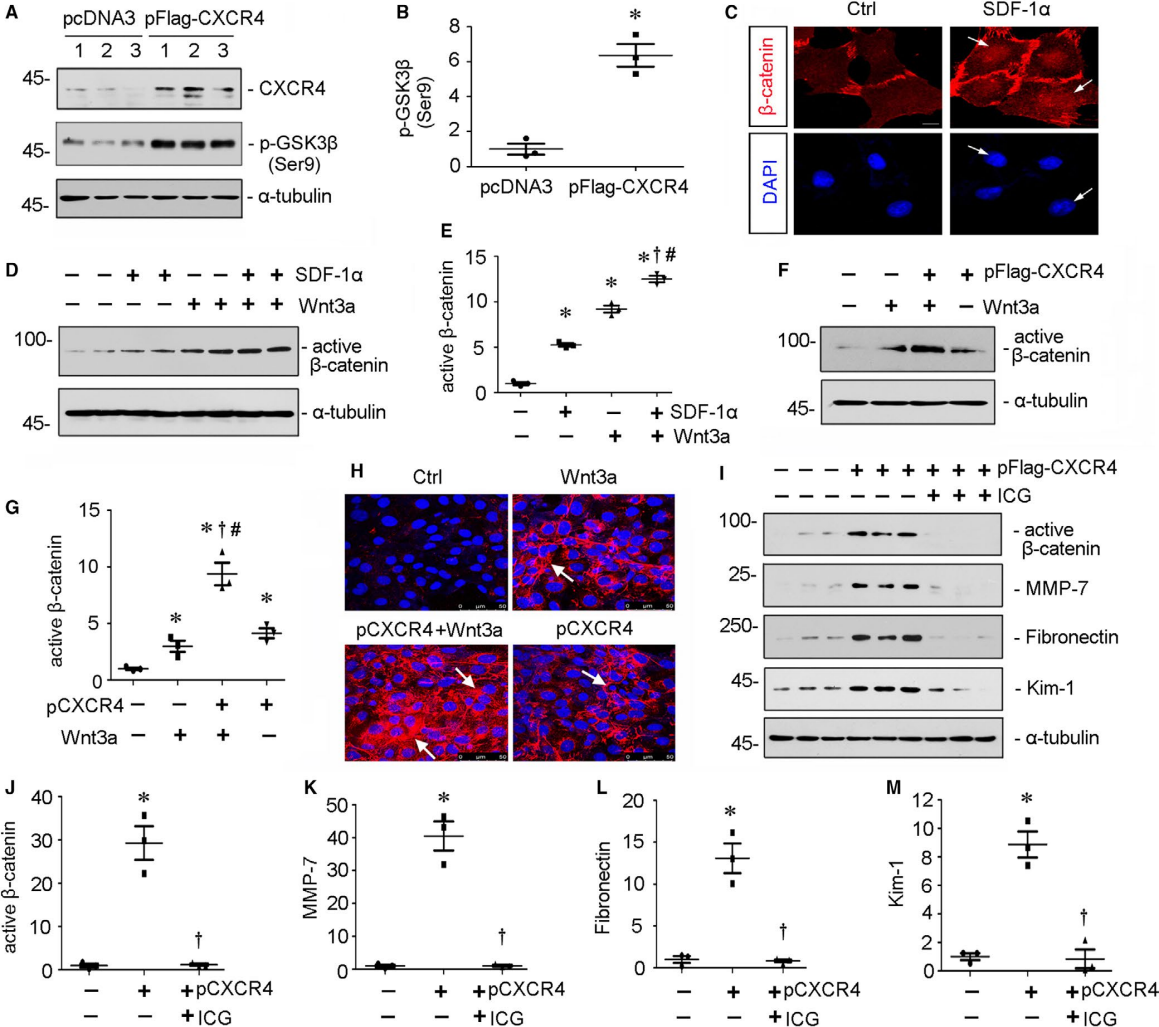
CXCR4/β‐catenin axis plays a critical role in tubular cell injury. A and B, Representative (A) Western blots and graphical representation of (B) p‐GSK3β (Ser9) protein expression in HKC‐8 cells. HKC‐8 cells were transfected with CXCR4 expression plasmid (pFlag‐CXCR4) or empty vector (pcDNA3) for 24 h. Whole cell lysates were analysed by Western blot analyses. Numbers 1‐3 represent each individual well in a given group. *P < .05 versus pcDNA3 group (n = 3). C, Representative micrographs show administration of SDF‐1α induced nuclear translocation of β‐catenin. HKC‐8 cells were treated with SDF‐1α (100 ng/mL) for 12 h. Cells were then stained for β‐catenin and DAPI, the nuclear marker. White arrows indicate positive nuclear staining. Scale bar, 10*µ*m. D and E, Representative (D) Western blots and graphical representation of (E) active β‐catenin protein expression in different groups. HKC‐8 cells were treated with SDF‐1α (100 ng/mL) and/or Wnt3a (100 ng/mL) for 12 h. *P < .05 versus control group (n = 3); †*P* < .05 versus Wnt3a alone (n = 3); ＃*P* < .05 versus SDF‐1α alone (n = 3). F and G, Representative (F) Western blots and graphical representation of (G) active β‐catenin protein expression in different groups. HKC‐8 cells were transfected with pFlag‐CXCR4 or pcDNA3 and then treated with or without Wnt3a (100 ng/mL) for 24 h. *P < .05 versus pcDNA3 group (n = 3); †*P* < .05 versus Wnt3a alone (n = 3); ＃*P* < .05 versus pCXCR4 (pFlag‐CXCR4) alone (n = 3). H, Representative micrographs show fibronectin expression (red) in four groups. HKC‐8 cells were transfected with pFlag‐CXCR4 or pcDNA3 plasmid, then treated with Wnt3a (100 ng/mL) for 24 h. Cells were then immunostained for fibronectin and counterstained with DAPI. White arrows indicate positive staining. I‐M, Representative (I) Western blots and graphical representations of (J) active β‐catenin, (K) MMP‐7, (L) fibronectin and (M) Kim‐1 in three groups. HKC‐8 cells were pretreated with ICG‐001 (5 µM) and then transfected with pFlag‐CXCR4 or pcDNA3 plasmid for 24 h. *P < .05 versus pcDNA3 group (n = 3); †*P* < .05 versus pCXCR4 (pFlag‐CXCR4) alone (n = 3)

To further figure out the important role of β‐catenin activation in CXCR4‐induced tubular injury, we performed analysis in a human proximal tubular cell line (HKC‐8). First, HKC‐8 cells were transfected with CXCR4 expression plasmid (pFlag‐CXCR4) or treated with SDF‐1α, the ligand of CXCR4. As shown in Figure 7A and B, overexpression of CXCR4 triggered the up‐regulation of p‐GSK3β (Ser9), a deactivated form of GSK3β which allows β‐catenin stabilization and activation. Furthermore, treatment with SDF‐1α induced the nuclear translocation of β‐catenin in HKC‐8 cells (Figure 7C), suggesting the induction of β‐catenin activation. We next treated HKC‐8 cells with SDF‐1α in the absence or presence of Wnt3a, a canonical Wnt which triggers activation of β‐catenin. As shown in Figure 7D and E, SDF‐1α and Wnt3a could induce the activation of β‐catenin, respectively. SDF‐1α significantly increased the expression of active β‐catenin after co‐culture with Wnt3a recombinant protein. Similar results were observed when HKC‐8 cells were transfected with CXCR4 expression plasmid and co‐cultured with Wnt3a (Figure 7F and G). Then, we checked fibrotic injury in HKC‐8 cells. The immunofluorescence staining of fibronectin showed that CXCR4 had a cooperative effect with Wnt3a in cell fibrotic injury (Figure 7H). Furthermore, we pretreated HKC‐8 cells with ICG‐001 and then transfected with pFlag‐CXCR4 plasmid. Western blot analyses indicated administration of ICG‐001 significantly inhibited the up‐regulation of active β‐catenin and its target MMP‐7 by ectopic expression of CXCR4 (Figure 7I‐K). Moreover, treatment with ICG‐001 also largely inhibited CXCR4‐induced up‐regulation of fibronectin and Kim‐1, a glycoprotein serving as a marker of tubular injury (Figure 7I, L and M).^32^ These findings strongly suggest that β‐catenin activation has a decisive role in meditating CXCR4 signalling.

### 3.8 JAK/STAT pathway mediates activation of β‐catenin in CXCR4 signalling

**Figure 8:**
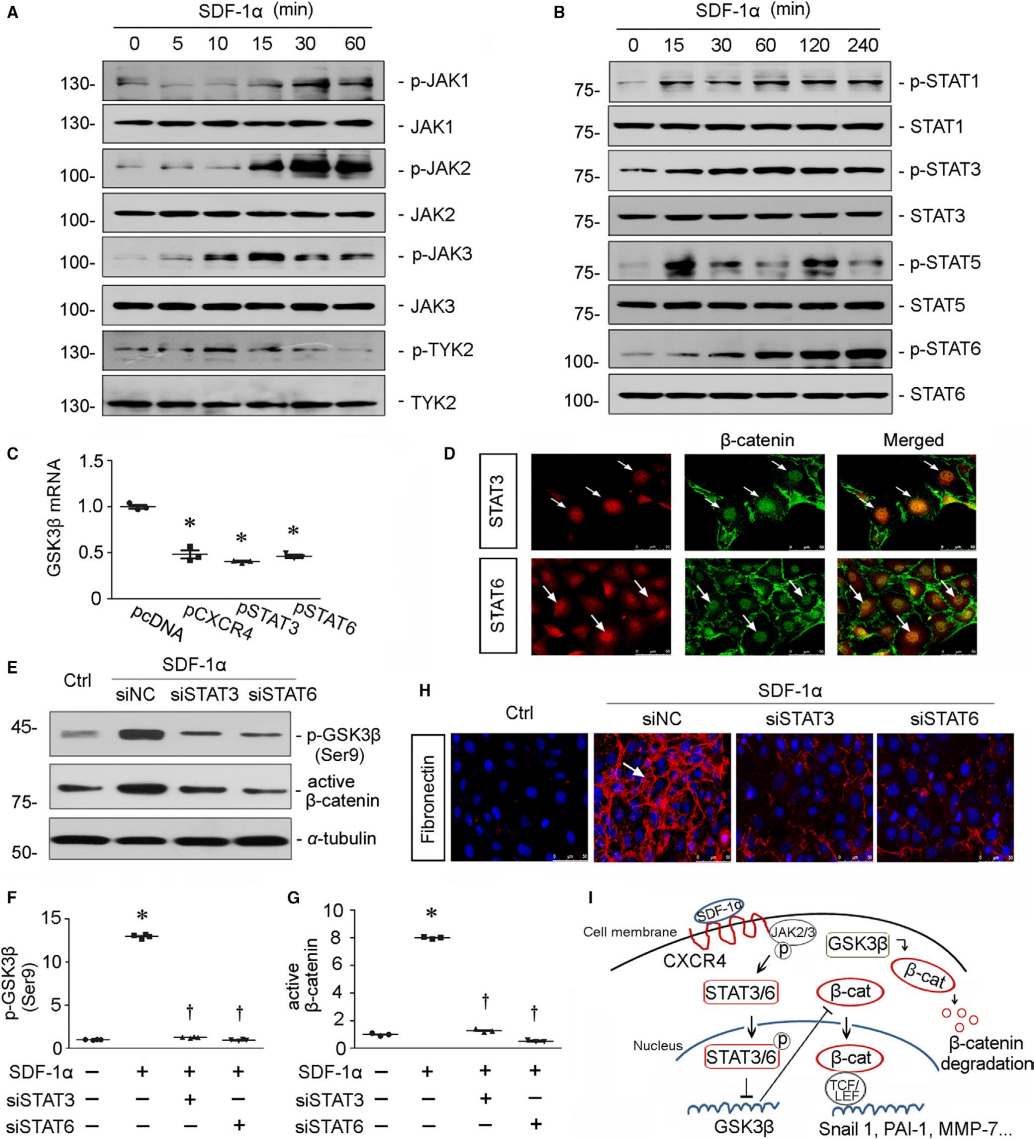
JAK/STAT pathway mediates β‐catenin activation in CXCR4 signalling. A, Representative Western blots show JAKs and p‐JAKs expression in HKC‐8 cells. HKC‐8 cells were incubated with SDF‐1α (100 ng/mL) for 0, 5, 10, 15, 30 and 60 min. B, Representative Western blots show STATs and p‐STATs expression in HKC‐8 cells. HKC‐8 cells were incubated with SDF‐1α (100 ng/mL) for 0, 15, 30, 60, 120 and 240 min. C, The relative abundance of GSK3β mRNA was assessed by quantitative real‐time PCR. HKC‐8 cells were transfected with CXCR4 or STAT3 or STAT6 expression plasmid (pFlag‐CXCR4, pFlag‐STAT3 or pHA‐STAT6) or pcDNA3 for 24 h. Total RNA was extracted and analysed by real‐time PCR. *P < .05 versus pcDNA3 group (n = 3). D, Representative micrographs show the colocalization of β‐catenin and STAT3 or STAT6 inHKC‐8 cells. HKC‐8 cells were treated with SDF‐1α (100 ng/mL) for 12 h. White arrows indicate the colocalization of β‐catenin and STAT3 or STAT6 in nuclei. E‐G, Representative (E) Western blots and graphical representations of (F) p‐GSK3β (Ser9) and (G) active β‐catenin expression in four groups. HKC‐8 cells were transfected with siRNA to STAT3 or STAT6 or negative control (NC) and then treated with SDF‐1α for 12 h.*P < .05 versus control group (n = 3 to 4); †*P* < .05 versus SDF‐1α plus NC group (n = 3 to 4). H, Representative micrographs show the expression of fibronectin in four groups as indicated. HKC‐8 cells were transfected with siRNA to STAT3 or STAT6 or NC and then treated with SDF‐1α for 12 h. Cells were then immunostained for fibronectin (red) and counterstained with DAPI (blue).White arrow indicates positive staining. I, Schematic presentation maps show that through binding of SDF‐1α to its receptor CXCR4, JAK2 and JAK3 are phosphorylated to trigger the activation and nuclear translocation of STAT3 and STAT6, which repress the transcription of GSK3β and the assembly of the β‐catenin degradation complex. The release of β‐catenin from degradation complex in the cytoplasm would lead to the accumulation of nuclear β‐catenin, which mediates fibrotic injury in tubular cells through binding to TCF/LEF transcription factors

It was previously reported that the cytoplasmic domain of CXCR4 protein involves in JAK2 and STAT3 phosphorylation.^33^ To further clarify the underlying mechanisms of CXCR4 in activation of β‐catenin, JAK/STAT pathway was studied in SDF‐1α‐treated HKC‐8 cells. As shown in Figure 8A and B, administration of SDF‐1α induced the phosphorylation of JAK2/STAT3 and JAK3/STAT6 in a time‐dependent manner, suggesting the potential role of JAK/STAT pathway in CXCR4 signalling. We next assessed the role of STAT3 or STAT6 in CXCR4‐induced β‐catenin signalling. HKC‐8 cells were transfected with CXCR4, STAT3 or STAT6 expression plasmid and then assessed the abundance of GSK3β mRNA. As shown in Figure 8C, ectopic expression of CXCR4, STAT3 or STAT6 could all significantly decrease the mRNA level of GSK3β. Furthermore, treatment with SDF‐1α induced the colocalization of β‐catenin with STAT3 or STAT6 (Figure 8D). Administration of SDF‐1α also induced the up‐regulation of p‐GSK3β (Ser9) and active β‐catenin protein expression (Figure 8E‐G). However, siRNA‐mediated inhibition of STAT3 or STAT6 blocked these effects in SDF‐1α‐treated cells. Similar results were observed when the expression of fibronectin was assessed by immunofluorescence staining (Figure 8H). These data undoubtedly demonstrate that JAK2/STAT3 and JAK3/STAT6 signalling play the important roles in CXCR4‐induced β‐catenin activation. Taken together, as summarized in Figure 8I, it is concluded that through the binding of SDF‐1α to CXCR4, JAK2 and JAK3 are phosphorylated to trigger the activation and nuclear translocation of STAT3 and STAT6, which repress the transcription of GSK3β and the assembly of the β‐catenin degradation complex. The release of β‐catenin from degradation complex in the cytoplasm would lead to the accumulation of nuclear β‐catenin, which mediates fibrotic injury in tubular cells through binding to transcription factors TCF/LEF.

### 3.9 Blockade of CXCR4 inhibits the activation of JAK/STAT in vivo

**Figure 9:**
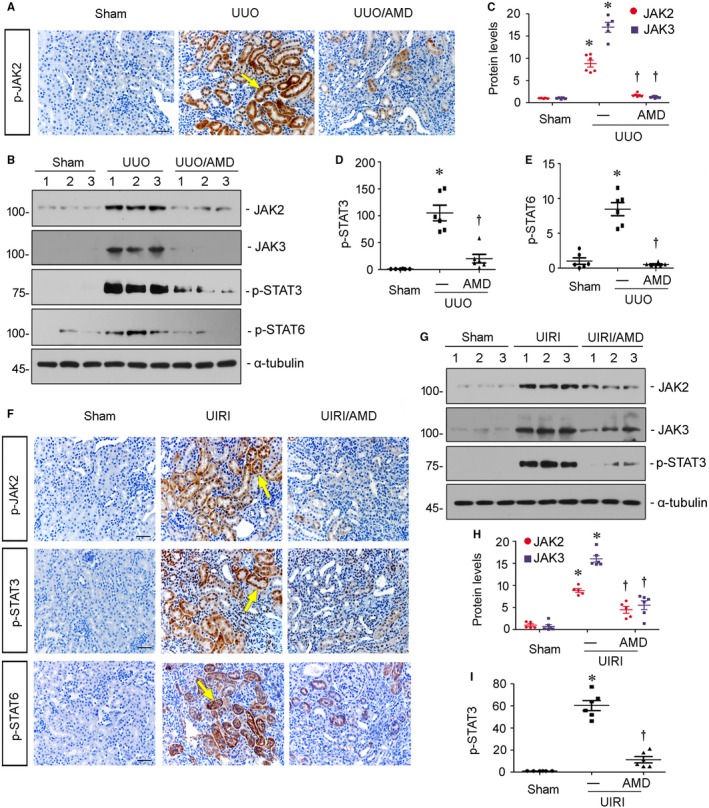
Blockade of CXCR4 inhibits JAK/STAT signalling in vivo. A, Representative micrographs show renal expression of p‐JAK2 in three groups. Paraffin kidney sections were immunostained with an antibody against p‐JAK2. Yellow arrow indicates positive staining. Scale bar, 50 μm. B‐E, Representative (B) Western blots and graphical representations of (C) JAK2 and JAK3, (D) p‐STAT3 and (E) p‐STAT6 in three groups. Numbers 1‐3 indicate each individual animal in a given group. *P < .05 versus sham control (n = 5‐6); †*P* < .05 versus UUO alone (n = 5‐6). F, Representative micrographs show renal expression of p‐JAK2, p‐STAT3 and p‐STAT6 in three groups. Paraffin kidney sections were immunostained with an antibody against p‐JAK2 or p‐STAT3 or p‐STAT6. Yellow arrows indicate positive staining. Scale bar, 50 μm. G‐I, Representative (G) Western blots and graphical representations of (H) JAK2 and JAK3, and (I) p‐STAT3 in different groups. Numbers 1‐3 indicate each individual animal in a given group. *P < .05 versus sham control (n = 5‐6); †*P* < .05 versus UIRI alone (n = 5‐6)

To further testify the modulation of JAK/STAT pathway by CXCR4, we next analysed the activity of JAK2/STAT3 and JAK3/STAT6 in UUO and UIRI mice administered AMD3100. The detailed experimental designs were presented in Figures 2A and 4A. As shown in Figure 9A, the expression of p‐JAK2 was evidently up‐regulated in renal tubular cells in UUO mice. Administration of AMD3100 greatly decreased the expression of p‐JAK2 in UUO mice. Similar results were observed when the protein expression levels of JAK2, JAK3, p‐STAT3 and p‐STAT6 were assessed by Western blot analyses (Figure 9B‐E). Consistently, in UIRI mice, administration of AMD3100 clearly diminished the up‐regulation of p‐JAK2, p‐STAT3 and p‐STAT6 (Figure 9F). Similar results were observed when the protein expression levels of JAK2, JAK3 and p‐STAT3 were assessed by Western blot analyses (Figure 9G‐I). These results further clarify the mediating roles of JAK/STAT in CXCR4 signalling.

### 3.10 Knockdown of CXCR4 blocks renal fibrosis through inhibition of JAK/STAT/GSK3β/β‐catenin pathway

**Figure 10:**
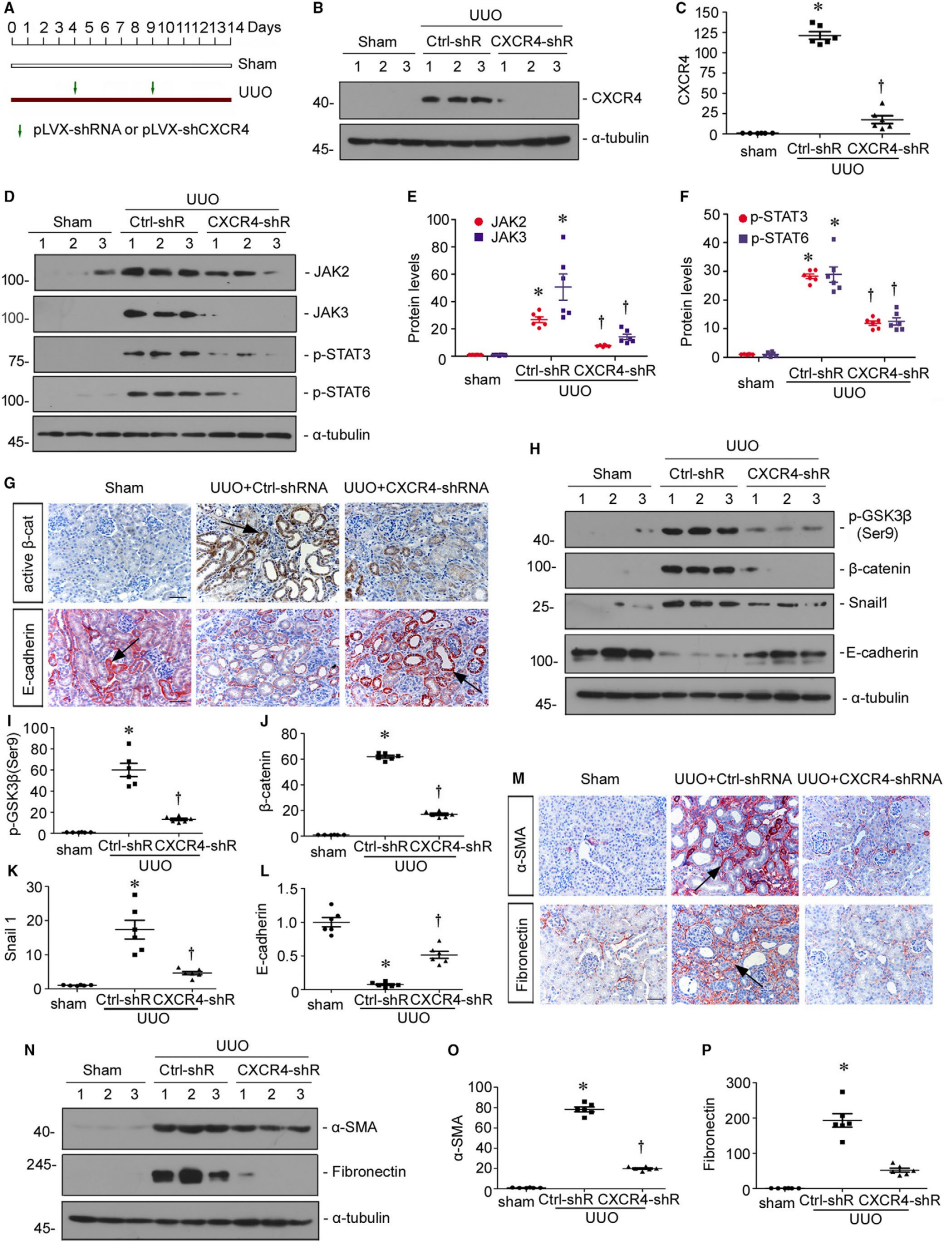
Knockdown of CXCR4 blocks renal fibrosis through inhibition of JAK/STAT/GSK3β/β‐catenin pathway. A, Experimental design. Green arrows indicate the injections of empty vector (pLVX‐shRNA) or an shRNA vector encoding the interference sequence of CXCR4 (pLVX‐shCXCR4). B and C, Representative (B) Western blots and graphical representation of (C) CXCR4 protein expression in three groups. Numbers 1‐3 indicate each individual animal in a given group. *P < .05 versus sham control mice (n = 6); †*P* < .05 versus UUO mice (n = 6). D‐F, Representative (D) Western blots and graphical representations of (E) JAK2 and JAK3, (F) p‐STAT3 and p‐STAT6 in three groups. Numbers 1‐3 indicate each individual animal in a given group. *P < .05 versus sham control mice (n = 6); †*P* < .05 versus UUO mice (n = 6). G, Representative micrographs show renal expression of active β‐catenin and E‐cadherin in three groups. Paraffin kidney sections were immunostained with an antibody against active β‐catenin or E‐cadherin. Black arrows indicate positive staining. Scale bar, 50 μm. active β‐cat, active β‐catenin. H‐L, Representative (H) Western blots and graphical representations of (I) p‐GSK 3β (Ser9), (J) β‐catenin, (K) Snail 1 and (L) E‐cadherin in three groups. Numbers 1‐3 indicate each individual animal in a given group. *P < .05 versus sham control mice (n = 6); †*P* < .05 versus UUO mice (n = 6). M, Representative micrographs show renal expression of α‐SMA and fibronectin in three groups. Paraffin kidney sections were immunostained with an antibody against α‐SMA or fibronectin. Black arrows indicate positive staining. Scale bar, 50 μm. N‐P, Representative (N) Western blots and graphical representations of (O) α‐SMA and (P) fibronectin in three groups. *P < .05 versus sham control mice (n = 6); †*P* < .05 versus UUO mice (n = 6)

To further prove the role of CXCR4 in renal fibrosis, we knocked down renal CXCR4 expression in vivo. The mice were injected with shRNA vector encoding the interference sequence of CXCR4 (pLVX‐shCXCR4) through a hydrodynamic‐based gene delivery approach. The detailed experimental design was shown in Figure 10A. The efficacy of CXCR4 knockdown was confirmed by Western blot analyses (Figure 10B and C). We then examined the expression of JAK/STAT signalling. As shown in Figure 10D‐F, knockdown of CXCR4 significantly decreased the activation of JAK2, JAK3, p‐STAT3 and p‐STAT6 in UUO mice. Consequently, the activation of p‐GSK3β (ser9), β‐catenin and Snail 1 induced in UUO mice were greatly prevented by knockdown of CXCR4 (Figure 10G‐K). In addition, the expression of E‐cadherin was restored by interference of CXCR4 in UUO mice (Figure 10G, H and L). We next assessed the fibrotic lesions. As shown in Figure 10M‐P, the expression levels of α‐SMA and fibronectin were induced in UUO mice, but significantly diminished by interference of CXCR4. These results further demonstrated that CXCR4 triggers renal fibrosis through induction of JAK/STAT/GSK3β/β‐catenin pathway.

## 4 DISCUSSION

More than 10% of the global populations are suffering from CKD. Unfortunately, a large portion of them would inevitably progress into the end‐stage renal disease (ESRD).^34^ Renal fibrosis is the common feature of morphologic changes in various types of CKD. However, because of the complicated mechanisms,^35^, ^36^ renal fibrosis is an intractable problem to nephrologists worldwide.

Renal fibrosis is initially characterized by the excessive matrix deposition in the interstitial area. With the progression of disease, the massive collagen deposition in the interstitial area could invade nearby nephron, the basic structural and functional unit of the kidney, and spread quickly. In humans with a variety of nephropathies and experimental CKD models, it is often seen that tubular cells may undergo cell injury in different ways with the secretion of fibrosis‐related molecules, especially in the late stage of disease.^37^, ^38^ Large numbers of reports show that tubular epithelial cells could transit into mesenchymal, dedifferentiated and senescent phenotypes to secret matrix‐synthesizing molecules such as TGF‐β1 and proinflammatory cytokines such as IL‐6.^18^, ^26^ Notably, TGF‐β1 and IL‐6 are the major mediators in interstitial fibroblast activation and macrophage bursting,^39^, ^40^ two key factors in the progression of renal fibrosis. These suggest tubular cell injury occupies a fundamental position in controlling the fate of CKD patients.^41^ Hence, to deeply understand the molecular mechanisms of tubular cell injury would undoubtedly provide new insights and strategies in the management of CKD.

C‐X‐C motif chemokine receptor (CXCR) family is a member of seven‐transmembrane receptors, which exerts chemoattractant effects on leucocytes and other cell types in controlling cell activation and guiding cell movements under basal and inflammatory conditions.^42^ There are 8 members of C‐X‐C motif chemokine receptors (CXCR1‐8), which play the critical roles in immune, neurodegenerative and cardiovascular diseases.^43^, ^44^, ^45^ Recent reports show that several C‐X‐C motif chemokine receptors also play the fundamental roles in the progression of CKD, such as CXCR1,^46^ CXCR6^47^ and particularly CXCR4.^7^, ^8^, ^10^ It was reported in our previous study, in glomeruli of CKD patients and mouse models, CXCR4 expression is up‐regulated predominantly in podocytes and is responsible for oxidative stress.^7^ Furthermore, in lupus nephritis patients, CXCR4 aggravates the severity of diseases through activating CD19 + B cells and CD4 + T cells.^9^, ^48^ Another report shows macrophage‐expressed CXCR4 aggravates renal fibrosis.^8^ In that study, they also found ablation of tubular CXCR4 attenuates renal fibrosis. However, the role of CXCR4 in tubular cell injury and the underlying mechanisms need to be elucidated in detail.

β‐catenin is the downstream effector of Wnt signalling in tubular cell senescence,^18^ podocyte dedifferentiation 15and fibroblast activation.^49^ Although β‐catenin expression is silent in normal adult kidneys, it is reactivated at an early stage and keeps activation in the progression of CKD.^50^, ^51^ β‐catenin could express in various cells; however, it is predominantly located in tubular cells in injured kidneys.^23^ Except Wnt signalling, β‐catenin also plays a functional role in the signal transduction of ET‐1 receptor, a member of G‐protein–coupled receptors (GPCRs).^52^ Interestingly, CXCR4 is a receptor that belongs to the superfamily of GPCRs. These prompt us to check the correlation between CXCR4 and β‐catenin.

We first assessed the expression of CXCR4 and β‐catenin in experimental mouse models. The results show that CXCR4 and β‐catenin were both up‐regulated predominantly in injured tubular cells in a time‐dependent manner. The results of staining on sequential sections further claim the co‐localization of CXCR4 and β‐catenin in tubular cells. Furthermore, the expression of CXCR4 has a positive correlation to the expression of MMP‐7 and triggers signal amplification of MMP‐7, the downstream target of β‐catenin (Figure 1). We also found in humans with IgAN, RPGN and FSGS, the up‐regulation of CXCR4 was mainly localized in tubular cells (Figure 1). Large amounts of recent reports have shown that β‐catenin is up‐regulated in tubular cells and plays a critical role in cell injury in these diseases.^53^, ^54^, ^55^ Hence, these data suggest that CXCR4 has a potential role in tubular cell injury and is associated with β‐catenin activation.

We next performed the analysis in experimental mouse models and in cultured tubular cells. In UUO and UIRI mice, blockade of CXCR4 by AMD3100 significantly inhibited the activation of p‐GSK3β/β‐catenin signalling, thereby protected against renal fibrotic lesions and preserved kidney functions (Figures 2, 3, 4, 5). We also testified the effects of ICG‐001, the inhibitor of β‐catenin signalling, on CXCR4‐aggravated renal fibrosis. Interestingly, administration of ICG‐001 could significantly block CXCR4‐induced renal fibrosis (Figure 6), further suggesting the mediating role of β‐catenin in CXCR4 cascade. The in vitro studies show that CXCR4 cooperatively acted with Wnt on β‐catenin signalling activation and fibrotic lesions (Figure 7). Meanwhile, SDF‐1α, the ligand of CXCR4, evidently induced the phosphorylation and activation of JAK2/STAT3 and JAK3/STAT6 (Figure 8), the two key effectors of signalling cascade in JAK/STAT pathways.^56^ Furthermore, overexpression of STAT3 or STAT6 repressed GSK3β transcription, and siRNA‐mediated inhibition of STAT3 or STAT6 could significantly inhibit SDF‐1α‐induced β‐catenin activation and fibrotic lesions (Figure 8). The mediating roles of JAK/STAT in CXCR4 signalling were also proved in AMD3100‐administered and CXCR4 gene‐silenced CKD mouse models (Figures 9 and 10). These data clearly demonstrate that β‐catenin signalling mediates CXCR4 cascade in tubular cell injury. The regulation of GSK3β by JAK/STAT pathway plays a central role in CXCR4‐induced β‐catenin activation. Consistently, it was reported that the cytoplasmic domain of CXCR4 protein could phosphorylate JAK2.^33^

One of the novel findings in the present study is that the transcription factors STAT3 and STAT6 could regulate GSK3β/β‐catenin signalling in renal tubular cells (Figure 8).This observation, for the first time, unequivocally links intercellular messenger's signals to β‐catenin activation, which would provide broad implications beyond tubular pathology. GSK3β is the serine/threonine protein kinase mediating β‐catenin degradation.^57^ Compared with the inhibitors of β‐catenin signalling such as ICG‐001,^58^ GSK3β inducers would undoubtedly provide unique inhibition to β‐catenin signalling, as it would trigger the degradation of β‐catenin other than the single inhibition of transcriptional activity. Additionally, although Wnt inhibitors such as DKK1 have inhibitory effects on activation of β‐catenin,^59^ however, the broad‐spectrum inhibition of multiple Wnts activity would certainly induce both good and bad effects as some Wnts may have the blocking effects on renal fibrosis.^60^, ^61^ Hence, to develop the therapeutics from the promoters of GSK3β would provide new insights to block the reactivated β‐catenin signalling in tubular cell injury and renal fibrosis. In motion, a clinical trial of JAK2 inhibitor in diabetic kidney diseases is ongoing with good signs for protecting efficacy.^62^ Although JAK/STAT pathways exert various functions in renal fibrosis,^63^, ^64^, ^65^, ^66^ we believe that GSK3β/β‐catenin signalling is an important downstream effector that should not to be ignored. We admit CXCR4 could mediate diverse signalling including JAK/STAT pathway,^67^ which also plays an important role in renal fibrosis.^68^ However, our studies explicitly establish a role of CXCR4 in activation of β‐catenin signalling. Furthermore, JAK/STAT signalling plays very important roles in activation of β‐catenin triggered by CXCR4. Our studies provide new insights into the intricate mechanisms and give potential implications in the treatment of renal fibrosis and CKD.

## CONFLICT OF INTEREST

The authors declared no competing interests.

## AUTHORS’ CONTRIBUTIONS

Yahong Liu, QijianFeng, Jinhua Miao, Qinyu Wu, Shan Zhou and WeiweiShen conducted the experiments and prepared the materials involved in this study. Lili Zhou conceived this study and its design and coordination. Lili Zhou, Fan FanHou, Youhua Liu, YanqiuFeng, Jinhua Miao and WeiweiShen contributed to the analysis and interpretation of the data. Lili Zhou and Yahong Liu drafted the manuscript. All authors read and approved the final manuscript.

## Data Availability

The data that support the findings of this study are available from the corresponding author upon reasonable request.

## References

[1] Malhotra R , Craven T , Ambrosius WT , et al. Effects of intensive blood pressure lowering on kidney tubule injury in CKD: A longitudinal subgroup analysis in SPRINT. Am J Kidney Dis. 2019;73(1):21‐30. 30291012 10.1053/j.ajkd.2018.07.015 PMC7325694

[2] Humphreys BD . Mechanisms of renal fibrosis. Annu Rev Physiol. 2018;80:309‐326. 29068765 10.1146/annurev-physiol-022516-034227

[3] Bansal N , Xie D , Sha D , et al. Cardiovascular events after new‐onset atrial fibrillation in adults with CKD: results from the chronic renal insufficiency Cohort (CRIC) Study. J Am Soc Nephrol. 2018;29(12):2859‐2869. 30377231 10.1681/ASN.2018050514 PMC6287862

[4] Berthoux F , Suzuki H , Mohey H , et al. Prognostic value of serum biomarkers of autoimmunity for recurrence of IgA nephropathy after kidney transplantation. J Am Soc Nephrol. 2017;28(6):1943‐1950. 28255003 10.1681/ASN.2016060670 PMC5461789

[5] Wang K , Kestenbaum B . Proximal tubular secretory clearance: a neglected partner of kidney function. Clin J Am Soc Nephrol. 2018;13(8):1291‐1296. 29490976 10.2215/CJN.12001017 PMC6086711

[6] Christelle F , Monika W , Julie N , et al. Lymphoid differentiation of hematopoietic stem cells requires efficient Cxcr4 desensitization. J Exp Med. 2017;214(7):2023‐2040. 28550161 10.1084/jem.20160806 PMC5502422

[7] Mo H , Wu Q , Miao J , et al. Chemokine receptor Type 4 plays a crucial role in mediating oxidative stress‐induced podocyte injury. Antioxid Redox Signal. 2017;27(6):345‐362. 27960539 10.1089/ars.2016.6758 PMC6435352

[8] Yuan A , Lee YS , Choi U , et al. Chemokine receptor Cxcr4 contributes to kidney fibrosis via multiple effectors. Am J Physiol Renal Physiol. 2015;308(5):F459‐F472. 25537742 10.1152/ajprenal.00146.2014 PMC4346747

[9] Zhao LD , Liang D , Wu XN , et al. Contribution and underlying mechanisms of CXCR4 overexpression in patients with systemic lupus erythematosus. Cell Mol Immunol. 2017;14(10):842‐849. 27665947 10.1038/cmi.2016.47 PMC5649106

[10] Lotan D , Sheinberg N , Kopolovic J . Expression of SDF‐1/CXCR4 in injured human kidneys. Pediatr Nephrol. 2008;23(1):71‐77. 17972109 10.1007/s00467-007-0648-2

[11] Ding M , Cui SY , LiC J , et al. Loss of the tumor suppressor Vhlh leads to upregulation of Cxcr4 and rapidly progressive glomerulonephritis in mice. Nat Med. 2006;12(9):1081‐1087. 16906157 10.1038/nm1460

[12] Takabatake Y , Sugiyama T , Kohara H , et al. The CXCL12 (SDF‐1)/CXCR4 axis is essential for the development of renal vasculature. J Am Soc Nephrol. 2009;20(8):1714‐1723. 19443644 10.1681/ASN.2008060640 PMC2723985

[13] Siddiqi FS , Chen LH , Advani SL , et al. CXCR4 promotes renal tubular cell survival in male diabetic rats: implications for ligand inactivation in the human kidney. Endocrinology. 2015;156(3):1121‐1132. 25549045 10.1210/en.2014-1650

[14] Wang YP , Zhou CJ , Liu YH . Wnt signaling in kidney development and disease. Prog Mol Biol Transl Sci. 2018;153:181‐207. 29389516 10.1016/bs.pmbts.2017.11.019 PMC6008255

[15] Zhou L , Chen X , Lu M , et al. Wnt/β‐catenin links oxidative stress to podocyte injury and proteinuria. Kidney Int. 2019;95(4):830‐845. 30770219 10.1016/j.kint.2018.10.032 PMC6431566

[16] Zhao Y , Wang C , Hong X , et al. Wnt/β‐catenin signaling mediates both heart and kidney injury in type 2 cardiorenal syndrome. Kidney Int. 2019;95(4):815‐829. 30770217 10.1016/j.kint.2018.11.021 PMC6431558

[17] Feng Y , Ren J , Gui Y , et al. Wnt/β‐catenin‐promoted macrophage alternative activation contributes to kidney fibrosis. J Am Soc Nephrol. 2018;29(1):182‐193. 29021383 10.1681/ASN.2017040391 PMC5748914

[18] Luo C , Zhou S , Zhou Z , et al. Wnt9a promotes renal fibrosis by accelerating cellular senescence in tubular epithelial cells. J Am Soc Nephrol. 2018;29(4):1238‐1256. 29440280 10.1681/ASN.2017050574 PMC5875944

[19] Goel S , Chin EN , Fakhraldeen SA , et al. Both LRP5 and LRP6 receptors are required to respond to physiological Wnt ligands in mammary epithelial cells and fibroblasts. J Biol Chem. 2012;287(20):16454‐16466. 22433869 10.1074/jbc.M112.362137 PMC3351289

[20] Peghaire C , Bats ML , Sewduth R , et al. Fzd7 (Frizzled‐7) expressed by endothelial cells controls blood vessel formation through Wnt/β‐catenin canonical signaling. Arterioscler Thromb Vasc Biol. 2016;36(12):2369‐2380. 27758766 10.1161/ATVBAHA.116.307926

[21] Zuo Y , Liu Y . New insights into the role and mechanism of Wnt/β‐catenin signalling in kidney fibrosis. Nephrology. 2018;4:38‐43. 10.1111/nep.13472 30298654

[22] Zhou L , Li Y , Hao S , et al. Multiple genes of the renin‐angiotensin system are novel targets of Wnt/β‐catenin signaling. J Am Soc Nephrol. 2015;26(1):107‐120. 25012166 10.1681/ASN.2014010085 PMC4279741

[23] Zhou L , Li Y , Zhou D , et al. Loss of Klotho contributes to kidney injury by derepression of Wnt/β‐catenin signaling. J Am Soc Nephrol. 2013;24(5):771‐785. 23559584 10.1681/ASN.2012080865 PMC3636797

[24] He W , Tan RJ , Li Y , et al. Matrix metalloproteinase‐7 as a surrogate marker predicts renal Wnt/β‐catenin activity in CKD. J Am Soc Nephrol. 2012;23(2):294‐304. 22095947 10.1681/ASN.2011050490 PMC3269179

[25] Liang J , Liu L , Xing D . Photobiomodulation by low‐power laser irradiation attenuates Abeta‐induced cell apoptosis through the Akt/GSK3β/β‐catenin pathway. Free Radic Biol Med. 2012;53(7):1459‐1467. 22917976 10.1016/j.freeradbiomed.2012.08.003

[26] Liu Y . New insights into epithelial‐mesenchymal transition in kidney fibrosis. J Am Soc Nephrol. 2010;21(2):212‐222. 20019167 10.1681/ASN.2008121226 PMC4554339

[27] Zhou L , Liu Y . Wnt/beta‐catenin signalling and podocyte dysfunction in proteinuric kidney disease. Nat Rev Nephrol. 2015;11(9):535‐545. 26055352 10.1038/nrneph.2015.88 PMC4869701

[28] Yuan Y , Chen Y , Zhang P , et al. Mitochondrial dysfunction accounts for aldosterone‐induced epithelial‐to‐mesenchymal transition of renal proximal tubular epithelial cells. Free Radic Biol Med. 2012;53(1):30‐43. 22608985 10.1016/j.freeradbiomed.2012.03.015

[29] Buonafine M , Martinez‐Martinez E , Jaisser F . More than a simple biomarker: the role of NGAL in cardiovascular and renal diseases. Clin Sci. 2018;132(9):909‐923. 29739822 10.1042/CS20171592

[30] Hong Q , Zhang L , Fu J , et al. LRG1 promotes diabetic kidney disease progression by enhancing TGF‐β‐induced angiogenesis. J Am Soc Nephrol. 2019;30(4):546‐562. 30858225 10.1681/ASN.2018060599 PMC6442349

[31] Nlandu‐Khodo S , Neelisetty S , Philips M , et al. TGF‐β and β‐catenin epithelial crosstalk exacerbates CKD. J Am Soc Nephrol. 2017;28(12):3490‐3503. 28701516 10.1681/ASN.2016121351 PMC5698068

[32] Schrõppel B , Krüger B , Walsh L , et al. Tubular expression of KIM‐1 does not predict delayed function after transplantation. J Am Soc Nephrol. 2010;21(3):536‐542. 20019169 10.1681/ASN.2009040390 PMC2831861

[33] Ahr B , Denizot M , Robert‐Hebmann V , et al. Identification of the cytoplasmic domains of CXCR4 involved in Jak2 and STAT3 phosphorylation. J Biol Chem. 2005;280(8):6692‐7700. 15615703 10.1074/jbc.M408481200

[34] Pearce N , Caplin B , Gunawardena N , et al. CKD of unknown cause: A global epidemic? Kidney Int Rep. 2019;4(3):367‐369. 30899862 10.1016/j.ekir.2018.11.019 PMC6409411

[35] Hobby GP , Karaduta O , Dusio GF , et al. Chronic kidney disease and the gut microbiome. Am J Physiol Renal Physiol. 2019;316(6):F1211‐F1217. 30864840 10.1152/ajprenal.00298.2018 PMC6620595

[36] Teasdale EJ , Leydon G , Fraser S , et al. Patients' experiences after CKD diagnosis: A meta‐ethnographical study and systematic review. Am J Kidney Dis. 2017;70(5):656‐665. 28764920 10.1053/j.ajkd.2017.05.019

[37] Qi R , Yang C . Renal tubular epithelial cells: the neglected mediator of tubulointerstitial fibrosis after injury. Cell Death Dis. 2018;9(11):1126. 30425237 10.1038/s41419-018-1157-x PMC6233178

[38] Zhang WR , Parikh CR . Biomarkers of acute and chronic kidney disease. Annu Rev Physiol. 2019;81:309‐333. 30742783 10.1146/annurev-physiol-020518-114605 PMC7879424

[39] Wang Q , He Z , Huang M , et al. Vascular niche IL‐6 induces alternative macrophage activation in glioblastoma through HIF‐2alpha. Nat Commun. 2018;9(1):559. 29422647 10.1038/s41467-018-03050-0 PMC5805734

[40] Meng XM , Nikolic‐Paterson DJ , Lan HY . TGF‐β: the master regulator of fibrosis. Nat Rev Nephrol. 2016;12(6):325‐338. 27108839 10.1038/nrneph.2016.48

[41] Dubin RF , Judd S , Scherzer R , et al. Urinary tubular injury biomarkers are associated with ESRD and death in the REGARDS study. Kidney Int Rep. 2018;3(5):1183‐1192. 30197985 10.1016/j.ekir.2018.05.013 PMC6127450

[42] Kufareva I , Gustavsson M , Zheng Y , et al. What Do structures tell us about chemokine receptor function and antagonism? Annu Rev Biophys. 2017;46:175‐198. 28532213 10.1146/annurev-biophys-051013-022942 PMC5764094

[43] Liu J , Zhang X , Chen K , et al. CCR7 chemokine receptor‐inducible lnc‐Dpf3 restrains dendritic cell migration by inhibiting HIF‐1alpha‐mediated glycolysis. Immunity. 2019;50(3):600‐15.e15. 30824325 10.1016/j.immuni.2019.01.021

[44] Wang Y , Dembowsky K , Chevalier E , et al. C‐X‐C motif chemokine receptor 4 blockade promotes tissue repair after myocardial infarction by enhancing regulatory T cell mobilization and immune‐regulatory function. Circulation. 2019;139(15):1798‐1812. 30696265 10.1161/CIRCULATIONAHA.118.036053 PMC6467561

[45] Krauthausen M , Kummer MP , Zimmermann J , et al. CXCR3 promotes plaque formation and behavioral deficits in an Alzheimer's disease model. J Clin Invest. 2015;125(1):365‐378. 25500888 10.1172/JCI66771 PMC4382235

[46] Swamydas M , Gao JL , Break TJ , et al. CXCR1‐mediated neutrophil degranulation and fungal killing promote Candida clearance and host survival. Sci Transl Med. 2016;8(322):322ra10. 10.1126/scitranslmed.aac7718 PMC4938152 26791948

[47] Xia Y , Yan J , Jin X , et al. The chemokine receptor CXCR6 contributes to recruitment of bone marrow‐derived fibroblast precursors in renal fibrosis. Kidney Int. 2014;86(2):327‐337. 24646857 10.1038/ki.2014.64 PMC4117803

[48] Wang A , Guiplain P , Chong BF , et al. Dysregulated expression of CXCR4/CXCL12 in subsets of patients with systemic lupus erythematosus. Arthritis Rheum. 2010;62(11):3436‐3446. 20722038 10.1002/art.27685 PMC8972909

[49] Zhou D , Fu H , Xiao L , et al. Fibroblast‐Specific β‐Catenin signaling dictates the outcome of AKL. J Am Soc Nephrol. 2018;29(4):1257‐1271. 29343518 10.1681/ASN.2017080903 PMC5875957

[50] Dai C , Stolz DB , Kiss LP , et al. Wnt/β‐catenin signaling promotes podocyte dysfunction and albuminuria. J Am Soc Nephrol. 2009;20(9):1997‐2008. 19628668 10.1681/ASN.2009010019 PMC2736766

[51] Xiao L , Zhou D , Tan RJ , et al. Sustained activation of Wnt/β‐catenin signaling drives AKI to CKD progression. J Am Soc Nephrol. 2016;27(6):1727‐1740. 26453613 10.1681/ASN.2015040449 PMC4884114

[52] Buelli S , Rosanò L , Gagliardini E , et al. β‐arrestin‐1 drives endothelin‐1‐mediated podocyte activation and sustains renal injury. J Am Soc Nephrol. 2014;25(3):523‐533. 24371298 10.1681/ASN.2013040362 PMC3935587

[53] Cox SN , Sallustio F , Serino G , et al. Altered modulation of WNT‐beta‐catenin and PI3K/Akt pathways in IgA nephropathy. Kidney Int. 2010;78(4):396‐407. 20485333 10.1038/ki.2010.138

[54] Tveita AA , Rekvig OP . Alterations in Wnt pathway activity in mouse serum and kidneys during lupus development. Arthritis Rheum. 2011;63(2):513‐522. 21280006 10.1002/art.30116

[55] Usui J , Kanemoto K , Tomari S , et al. Glomerular crescents predominantly express cadherin‐catenin complex in pauci‐immune‐type crescentic glomerulonephritis. Histopathology. 2003;43(2):173‐179. 12877733 10.1046/j.1365-2559.2003.01660.x

[56] Stabile H , Scarno G , Fionda C , et al. JAK/STAT signaling in regulation of innate lymphoid cells: The gods before the guardians. Immunol Rev. 2018;286(1):148‐159. 30294965 10.1111/imr.12705 PMC6178832

[57] Wu H , Lu XX , Wang JR , et al. TRAF6 inhibits colorectal cancer metastasis through regulating selective autophagic CTNNB1/β‐catenin degradation and is targeted for GSK3B/GSK3β‐mediated phosphorylation and degradation. Autophagy. 2019;15(9):1506‐1522. 30806153 10.1080/15548627.2019.1586250 PMC6693460

[58] Grigson ER , Ozerova M , Pisklakova A , et al. Canonical Wnt pathway inhibitor ICG‐001 induces cytotoxicity of multiple myeloma cells in Wnt‐independent manner. PLoS ONE. 2015;10(1):e0117693. 25635944 10.1371/journal.pone.0117693 PMC4311909

[59] He W , Dai C , Li Y , et al. Wnt/β‐catenin signaling promotes renal interstitial fibrosis. J Am Soc Nephrol. 2009;20(4):765‐776. 19297557 10.1681/ASN.2008060566 PMC2663839

[60] Lin SL , Li B , Rao S , et al. Macrophage Wnt7b is critical for kidney repair and regeneration. Proc Natl Acad Sci USA. 2010;107(9):4194‐4199. 20160075 10.1073/pnas.0912228107 PMC2840080

[61] Zhou D , Tan RJ , Fu H , Liu Y. Wnt/β‐catenin signaling in kidney injury and repair: a double‐edged sword. Lab Invest. 2016;96(2):156‐167. 26692289 10.1038/labinvest.2015.153 PMC4731262

[62] Brosius FC , Tuttle KR , Kretzler M . JAK inhibition in the treatment of diabetic kidney disease. Diabetologia. 2016;59(8):1624‐1627. 27333885 10.1007/s00125-016-4021-5 PMC4942738

[63] Yamada K , Huang ZQ , Raska M , et al. Inhibition of STAT3 signaling reduces IgA1 autoantigen production in IgA nephropathy. Kidney Int Rep. 2017;2(6):1194‐1207. 29270528 10.1016/j.ekir.2017.07.002 PMC5733772

[64] Edwards LJ , Mizui M , Kyttaris V . Signal transducer and activator of transcription (STAT) 3 inhibition delays the onset of lupus nephritis in MRL/lpr mice. Clin Immunol. 2015;158(2):221‐230. 25869298 10.1016/j.clim.2015.04.004 PMC4465043

[65] Takakura A , Nelson EA , Haque N , et al. Pyrimethamine inhibits adult polycystic kidney disease by modulating STAT signalling pathways. Hum Mol Genet. 2011;20(21):4143‐4154. 21821671 10.1093/hmg/ddr338 PMC3188991

[66] Talbot JJ , Shilingford JM , Vasanth S , et al. Polycystin‐1 regulates STAT activity by a dual mechanism. Proc Natl Acad Sci USA. 2011;108(19):7985‐7990. 21518865 10.1073/pnas.1103816108 PMC3093515

[67] Pawig L , Klasen C , Weber C , et al. Diversity and inter‐connections in the CXCR4 chemokine receptor/ligand family: molecular perspectives. Front Immunol. 2015;6:249. 26347749 10.3389/fimmu.2015.00429 PMC4543903

[68] Liu J , Zhong Y , Liu G , et al. Role of Stat3 signaling in control of EMT of tubular epithelial cells during renal fibrosis. Cell Physiol Biochem. 2017;42:2552. 28848189 10.1159/000480216

