# Corrigendum to Yahong Liu, Qijian Feng, Jinhua Miao, et al. ‘C‐X‐C motif chemokine receptor 4 aggravates renal fibrosis through activating JAK/STAT/GSK3β/β‐catenin pathway’ *Journal of Cellular and Molecular Medicine*. 2020;24(7):3837–3855

**DOI:** 10.1111/jcmm.17668

**Published:** 2023-04-12

In the above‐stated article, the authors mistakenly placed a duplicated images of Collagen I staining for the UIRI and UIRI/AMD groups in Figure 4K as same as the images of Collagen I staining for the UUO and UUO/AMD groups in Figure 2K. The authors would like to correct Figure 4K as following. Figure 4K now contains the correct Collagen I staining images.

**FIGURE 4:**
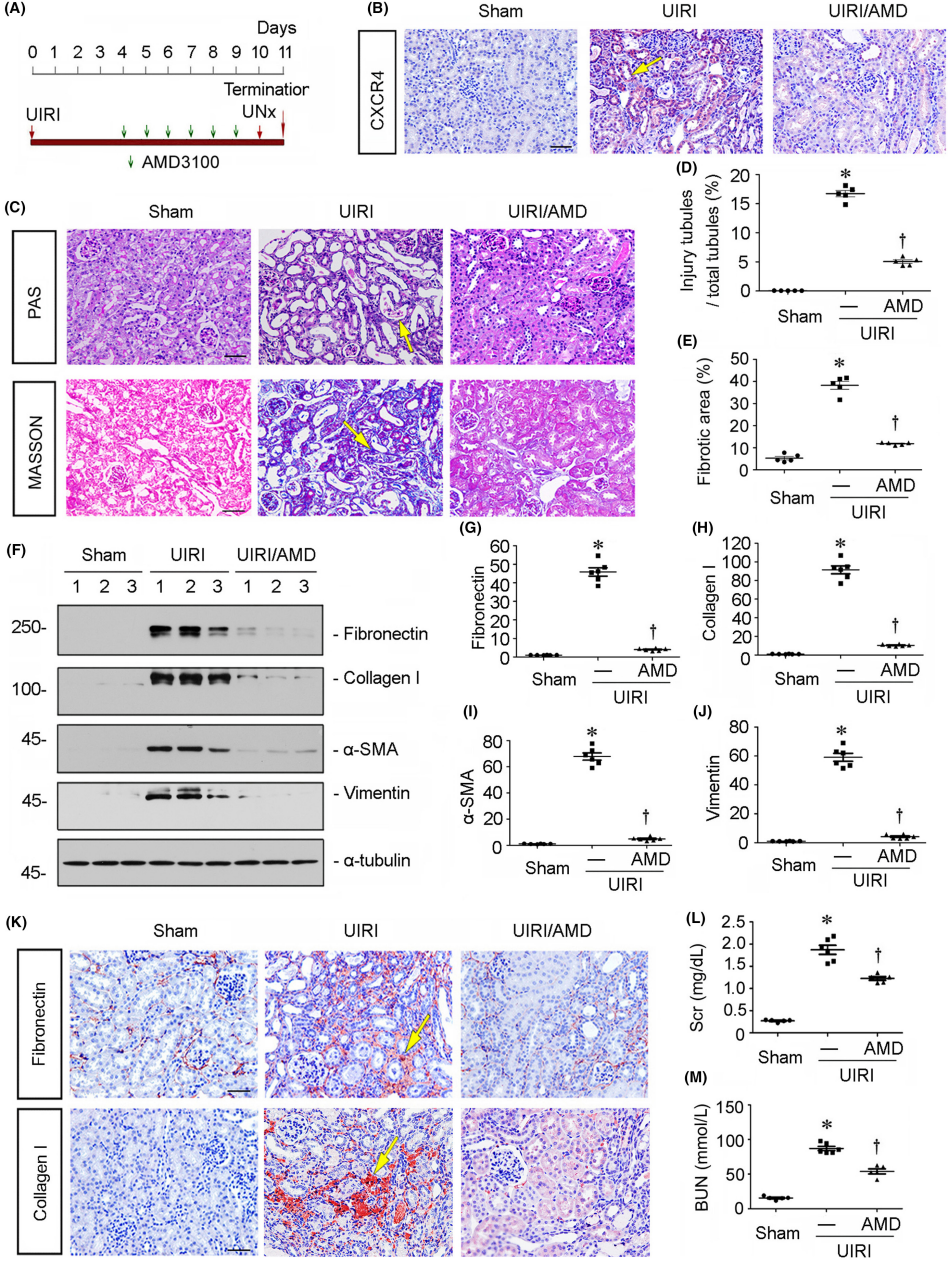
Blockade of CXCR4 mitigates renal fibrosis in UIRI mice. (A) Experimental design. Green arrows indicate the injections of AMD3100. Red arrows indicate the timing of renal IRI surgery. (B) Representative micrographs show renal expression of CXCR4 in different groups. Paraffin kidney sections were immunostained with an antibody against CXCR4. Arrow indicates positive staining. Scale bar, 50 μm. (C) Representative micrographs show PAS and Masson trichrome staining in different groups. Arrows indicate positive staining. Scale bar, 50 μm. (D and E) Graphical representations of quantitative analyses of (D) injured tubules and (E) renal fibrotic lesions in three groups. Kidney sections were subjected to PAS and Masson trichrome staining. At least 20 randomly selected fields were evaluated under ×400 magnification, and results were averaged for each kidney. *p < 0.05 versus sham control. †*p* < 0.05 versus UIRI alone (*n* = 5). (F–J) Representative (F) Western blots and graphical representations of (G) fibronectin, (H) collagen I, (I) α‐SMA and (J) vimentin protein expression levels in three groups. Numbers 1–3 indicate each individual animal in given group. *p < 0.05 versus sham control (*n* = 5–6); †*p* < 0.05 versus UIRI alone (*n* = 5–6). Scale bar, 50 μm. (K) Representative micrographs show renal expression of fibronectin and collagen I in three groups. Paraffin kidney sections were immunostained with antibodies against fibronectin and collagen I. Yellow arrows indicate positive staining. Scale bar, 50 μm. (L and M) Quantitative analysis of (L) serum creatinine (Scr) and (M) blood urea nitrogen (BUN) levels in three groups as indicated. *p < 0.05 versus sham control (*n* = 5–6); †*p* < 0.05 versus UIRI alone (*n* = 5–6). Scr was expressed as milligrams per decilitre, and BUN was expressed as millimole per litre

The authors would like to apologize for any inconvenience caused.

