# Supplementary figures and images for: C‐X‐C motif chemokine receptor 4 aggravates renal fibrosis through activating JAK/STAT/GSK3β/β‐catenin pathway
Source: J Cell Mol Med. 2020 Mar 2;24(7):3837–55. doi: 10.1111/jcmm.14973 (PMC7171406; doi:10.1111/jcmm.14973)

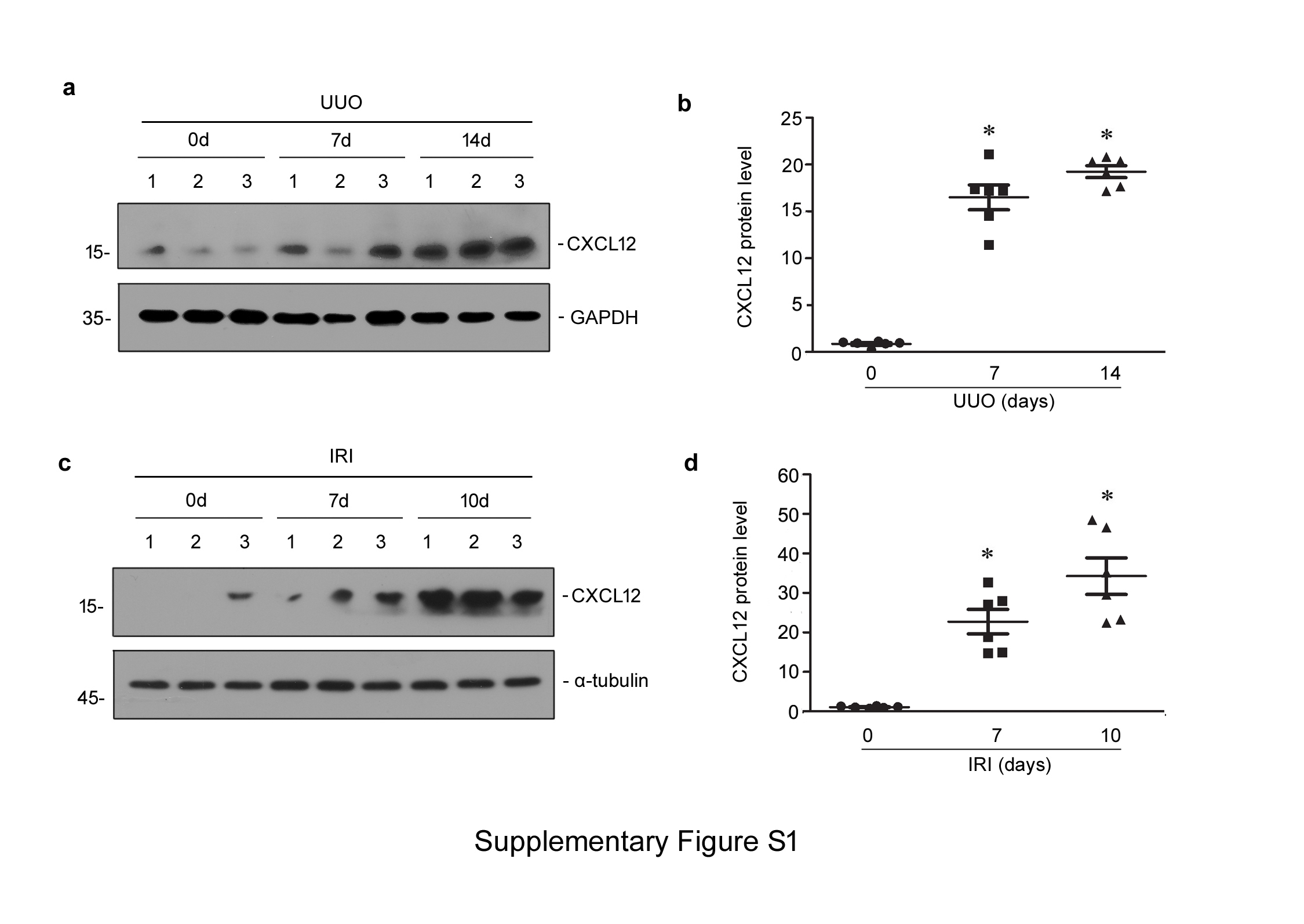

Supplement: Supplementary file 1 [file JCMM-24-3837-s001.jpg]

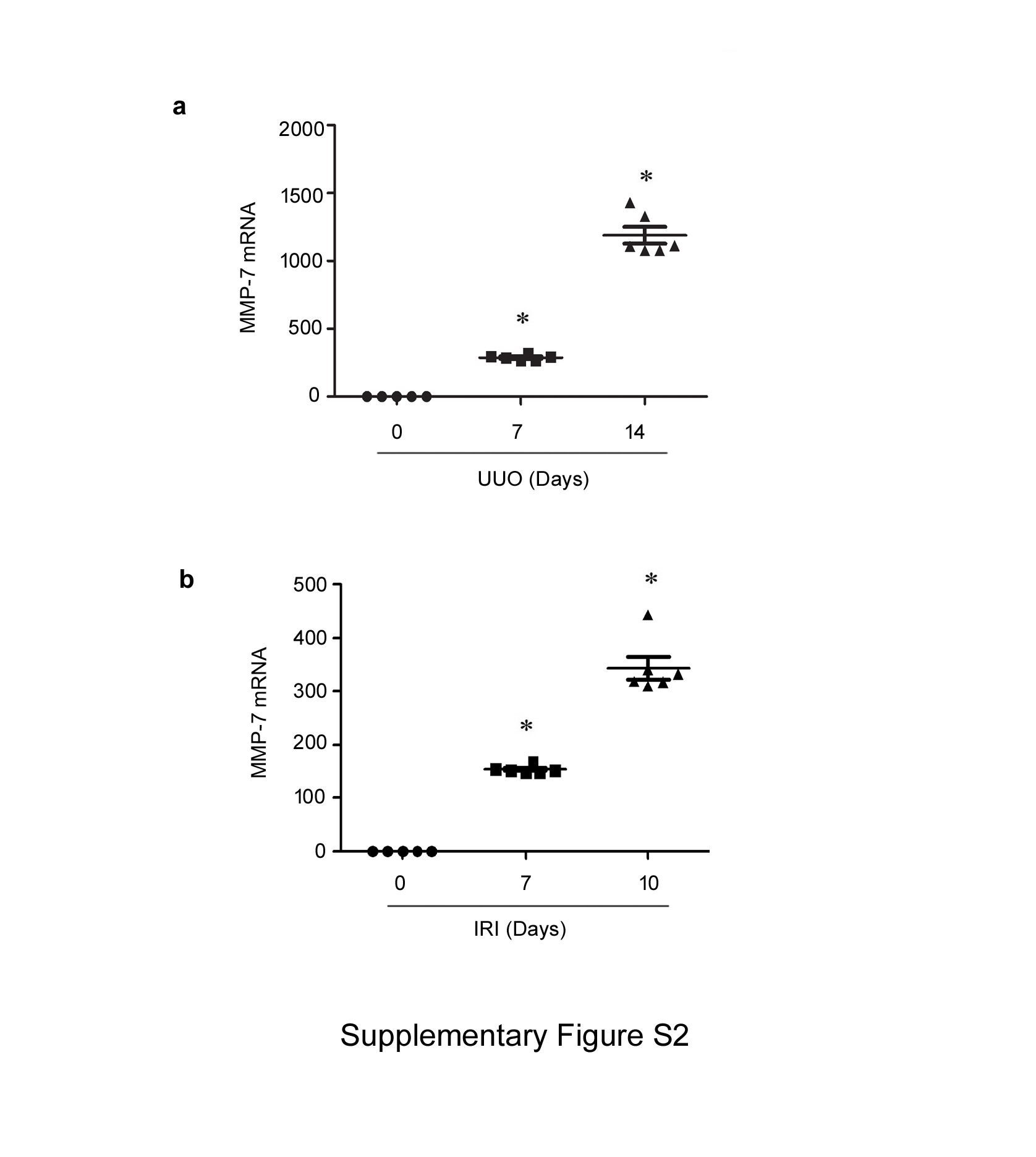

Supplement: Supplementary file 2 [file JCMM-24-3837-s002.jpg]
