# Supplementary material for: C‐X‐C motif chemokine receptor 4 aggravates renal fibrosis through activating JAK/STAT/GSK3β/β‐catenin pathway
Source: J Cell Mol Med. 2020 Mar 2;24(7):3837–55. doi: 10.1111/jcmm.14973 (PMC7171406; doi:10.1111/jcmm.14973)
Supplement: Supplementary file 3 [file JCMM-24-3837-s003.docx]

Supplementary Table 1. Nucleotide sequences of the primers used for PCR

| **Gene** | **Primer Sequence 5’ to 3’** | | **Product Size (bp)** |
| --- | --- | --- | --- |
|  | **Forward** | **Reverse** |  |
| **humanGSK3β** | **5'-GTCCGACTGCGGTATTTCTTC-3'** | **5'-CTCGATGGCAGATTCCAAAGG-3'** | **213** |
| **mouse CXCR4** | **5'-GACTGGCATAGTCGGCAATG-3'** | **5'-AGAAGGGGAGTGTGATGACAAA-3'** | **130** |
| **mouse MMP-7** | **5'-AGACAGCTTCCCCTTTGATGG-3'** | **5'-CAAATTCATGGGTGGCAGCAA-3'** | **158** |
| **mouse NGAL** | **5'-CTTGATCCCTGCCCCATCTC-3'** | **5'-ACATCGTAAAGCTGCCTTCTG-3'** | **137** |
| **mouse TGF-β1** | **5'-GTGGAAATCAACGGGATCAG-3'** | **5'-GTTGGTATCCAGGGCTCTCC-3'** | **150** |
| **human actin** | **5'-CTCACCATGGATGATGATATCGC-3'** | **5'-AGGAATCCTTCTGACCCATGC-3'** | **163** |
| **mouse actin** | **5'-GAGCGCAAGTACTCTGTGTG-3'** | **5'-AACGCAGCTCAGTAACAGTC-3'** | **152** |
